# Insights into the Effects of Cancer Associated Mutations at the UPF2 and ATP-Binding Sites of NMD Master Regulator: UPF1

**DOI:** 10.3390/ijms20225644

**Published:** 2019-11-11

**Authors:** Umesh Kalathiya, Monikaben Padariya, Kamila Pawlicka, Chandra S. Verma, Douglas Houston, Ted R. Hupp, Javier Antonio Alfaro

**Affiliations:** 1 International Centre for Cancer Vaccine Science, University of Gdansk, Wita Stwosza 63, 80-308 Gdansk, Poland; monika.padariya@pg.edu.pl; 2 Institute of Genetics and Molecular Medicine, University of Edinburgh, Edinburgh, Scotland EH4 2XR, UK; kamis125@gmail.com; 3 Bioinformatics Institute, Agency for Science, Technology and Research (A*STAR), 30 Biopolis Street, 07-01 Matrix, Singapore 138671, Singapore; chandra.verma@gmail.com; 4 Department of Biological Sciences, National University of Singapore, 16 Science Drive 4, Singapore 117558, Singapore; 5 School of Biological Sciences, Nanyang Technological University, 60 Nanyang Drive, Singapore 637551, Singapore; 6 Institute of Quantitative Biology, Biochemistry and Biotechnology, University of Edinburgh, Edinburgh, Scotland EH9 3BF, UK; douglasr.houston@ed.ac.uk

**Keywords:** UPF1, ATP-binding site, UPF2, cancer mutations, structural stability, molecular dynamics simulations

## Abstract

Nonsense-mediated mRNA decay (NMD) is a quality control mechanism that recognizes post-transcriptionally abnormal transcripts and mediates their degradation. The master regulator of NMD is UPF1, an enzyme with intrinsic ATPase and helicase activities. The cancer genomic sequencing data has identified frequently mutated residues in the CH-domain and ATP-binding site of UPF1. In silico screening of UPF1 stability change as a function over 41 cancer mutations has identified five variants with significant effects: K164R, R253W, T499M, E637K, and E833K. To explore the effects of these mutations on the associated energy landscape of UPF1, molecular dynamics simulations (MDS) were performed. MDS identified stable H-bonds between residues S152, S203, S205, Q230/R703, and UPF2/AMPPNP, and suggest that phosphorylation of Serine residues may control UPF1-UPF2 binding. Moreover, the alleles K164R and R253W in the CH-domain improved UPF1-UPF2 binding. In addition, E637K and E833K alleles exhibited improved UPF1-AMPPNP binding compared to the T499M variant; the lower binding is predicted from hindrance caused by the side-chain of T499M to the docking of the tri-phosphate moiety (AMPPNP) into the substrate site. The dynamics of wild-type/mutant systems highlights the flexible nature of the ATP-binding region in UPF1. These insights can facilitate the development of drug discovery strategies for manipulating NMD signaling in cell systems using chemical tools.

## 1. Introduction

Non-sense mediated mRNA decay (NMD) is a quality-control checkpoint that detects and eliminates aberrant messenger RNAs (mRNAs) with premature termination codons (PTCs) [1,2,3]. As such, the NMD complex can modulate the phenotypic outcome of genetic disorders caused by PTCs. Several studies have identified the existence of a transient ribosome associated complex containing eukaryotic release factor 1 (eRF1)-eRF3, up frameshift 1 (UPF1), ATP-dependent RNA helicase DEAH box polypeptide 34 (DHX34), and suppressor with morphological effect on genitalia 1 complex (SMG1C; containing SMG1 and its regulators SMG8 and SMG9) [4,5,6,7,8,9]. DHX34 dissociates DHX34-UPF1-SMG1C from ribosome-associated eRF1-eRF3 and promotes its association with UPF2-UPF3-EJC (exon junction complex) [5,9]. This may result in translation termination leading to the dissociation of the individual ribosomal subunits, release factors, and nascent protein. The UPF2 protein binds with the N-terminal domain of the UPF1 protein, and releases this domain from the central core of UPF1 [9,10,11].

Several additional subunits in the NMD complex have relatively well-defined functions. The SMG1 protein has a kinase domain that phosphorylates the UPF1 residues from the N- and C-terminal domains, and recruits the factors which initiate mRNA degradation by endonuclease cleavage (by SMG6), deadenylation (by the carbon catabolite repressor protein 4 (CCR4)-NOT deadenylase complex via the SMG5-SMG7) and/or decapping (by the decapping complex (DCPC) via Pro-rich nuclear receptor co-activator 2 (PNRC2)). UPF1 recruits DCPC independently of phosphorylation, and it has been suggested that recruitment of factors to the UPF1 phosphorylated C-terminal domain exclude this domain from the central core of UPF1 [9,12]. In addition, endonucleolytic cleavage, decapping, and deadenylation are sequentially carried out by complete mRNA degradation through the general cellular 5′–3′ and 3′–5′ exonucleolytic activities.

**Figure 1:**
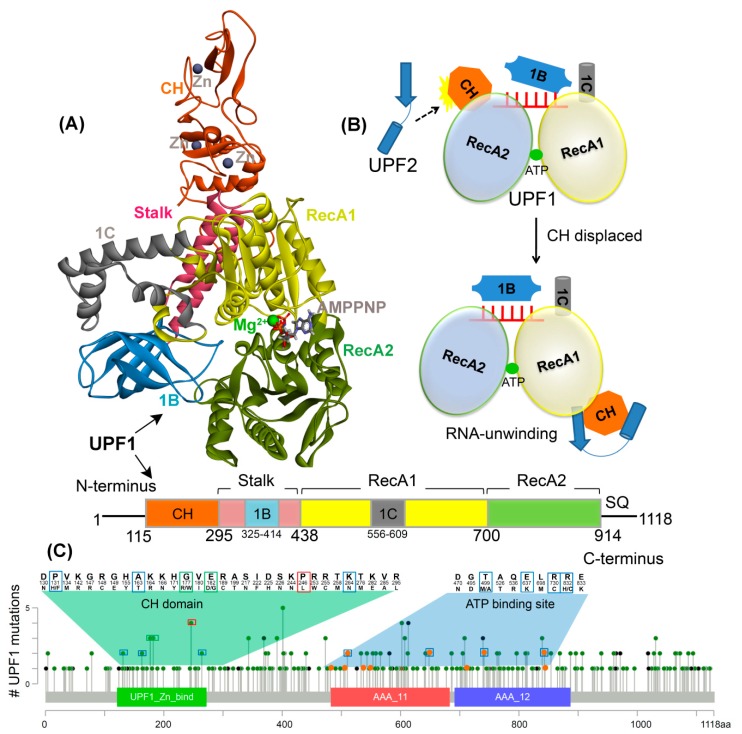
UPF1 structure and mutations from different cancer types. (**A**) Crystal structure of UPF1 (PDB: 2WJY [10]) representing different domains, ATP analogue (i.e., AMPPNP), and Zn/Mg^2+^ ions. (**B**) Schematic model of UPF1 activation mechanism showing how the RNA binding and ATPase/unwinding activity are modulated by UPF2 in an NMD cycle. In the absence of UPF2, the UPF1 has extended RNA interactions via the RecA1, RecA2, 1B, and 1C domains and low RNA-unwinding properties. The CH-domain is displaced upon UPF2 binding (black dotted arrow) and domain 1B changes to a conformation where it does not clamp on the RNA 3′ end and as a result, the RNA-unwinding activity is increased [11]. (**C**) UPF1 gene mutated in cancer, data retrieved from the cBioPortal database [29]. The frequency of a residue mutation equal to 2, 3, and 4 are marked in blue, green, and red boxes respectively. The mutations from the ATP-binding site are shown in orange. Color scheme: Zn and carbon in grey, Mg in green, oxygen in red, hydrogen in silver, nitrogen in blue, and sulfur in orange.

Three proteins, UPF1, UPF2, and UPF3 constitute the core of this NMD complex and are highly conserved across species. UPF1, an RNA-dependent ATPase is the master regulator of NMD and its ability to selectively target PTC-containing mRNAs depends on its ATPase and helicase activities [4,13,14,15,16], and this function is regulated through phosphorylation of its N- and C-terminal domains [17,18]. At the structural level, UPF1 contains a modular domain organization, with a conserved cysteine-histidine-rich domain (CH-domain), followed by superfamily 1 (SF1) helicase region that includes two RecA-like domains (RecA1 and RecA2), which comprise the motor of the helicase [19,20,21]. The CH-domain is stabilized by three zinc atoms bound by zinc finger-like motifs and the architecture of the CH-domain provides two distinct interfaces that bind to UPF2 (Figure 1A,B) [10,22]. Biochemical and structural analysis reveal that the complex of UPF2/UPF3 binds to the CH-domain and causes a large conformational change, activating the ATPase or helicase activity of the UPF1 protein [11].

Biochemical analysis and structural studies of the UPF1-RNA complex highlight four important aspects in its dynamic function [11]: (i) specific interactions occur in the presence or absence of the CH-domain that drive either a closed or open conformation of UPF1; (ii) the mechanical relationship between UPF1-catalyzed ATP-hydrolysis and the UPF1 translocation along RNA has been analyzed; (iii) the crystal structures of UPF1 in a closed conformation have shown that the CH-domain does not directly interact with RNA, but rather induces the positioning of other domains of UPF1 towards the binding site; (iv) UPF2 protein binding to the CH-domain of UPF1 stimulates the helicase activity of the latter by triggering a large scale conformational change of the CH-domain and by disrupting the CH- and helicase-domain interactions (closed conformation), thus promoting an open conformation of UPF1 [11].

Single amino acid mutations, residue changes, or single post-translational modifications such as the addition of phosphate or methyl groups at key sites within a protein may result in conformational changes, alterations of interaction networks, and hydrogen bond disruption, while these in turn may perturb the energy landscape. Moreover, mutation of an individual residue can affect or alter the kinetics of protein folding or cause protein aggregation and destabilization [23,24,25,26]. More than half of monogenic diseases are caused by single amino acid mutations and a common mechanism by which mutations cause human disease is protein stability change. This can result in enhanced or decreased protein degradation, mislocalization, or pathological re-wiring of its normal network by affecting protein–protein interactions. Approximately 88% of disease-associated non-synonymous single nucleotide polymorphisms (nsSNPs) are found to be located in the pockets important for protein–protein interactions [23,27,28].

The mutations located at the protein–protein binding interface could block the active site cleft, change binding affinity, and affect the structure of the protein complex. Therefore, in this study we focus on understanding the mutational landscape of the UPF1 protein in the structural context. Particularly, we explore the structural impact of these mutations on UPF1 regions including the CH-domain (important for UPF1-UPF2 binding) and the ATP-binding site (Figure 1). Previous studies have shown that disruption in the CH-domain could interfere with allosteric regulation [11]. In addition, simultaneous binding of UPF1 with ATP and RNA results in nucleotide-induced conformational changes in the ATP-binding and hydrolysis cycle, and the CH-domain regulates the extent of RNA binding by the helicase [11]. For the current study, we chose a subset of cancer associated mutations in the UPF1 protein from the cancer genomics database cBioPortal [29], and selected mutations that are located in the ATP-binding site or CH-domain of UPF1. Understanding how the mutations affect the stability of the UPF1 protein, and its interactions with ATP or with other proteins can provide new insights into the molecular mechanisms of this complex process and assist in drug discovery.

An examination of the mutational landscape of UPF1 in cBioPortal [29] reveals a broad spectrum of mutations occurring in this protein in a range of cancer types (Table S1); the highest frequency is observed in endometrial and ovarian cancers. A key challenge is to understand which ones, among all these mutations, are likely to have a functional impact. Although the frequency of mutated sites or regions impacted can be used to classify the mutations as drivers, many mutations discovered so far are observed in a relatively small fraction of tumors [30]. In our study, we integrated computational methods that can predict the effects of variations on protein structure stability or interactions, and can help to identify functionally important mutations. In addition, those mutations showing considerable changes to structural stability were further studied by molecular dynamics simulations (MDS) as protein structures undergo a plethora of different motions that can be perturbed by post-translational modifications, mutations, or interactions with binding partners [31]. To investigate these biologically meaningful dynamic processes, MDS enables an atomic level description of the UPF1 protein conformations, typically difficult to be accessible through the static structures from X-ray crystallography. This MDS-enabled dynamic perspective may contribute to the elucidation of the associated molecular mechanisms.

## 2. Results

### 2.1. Effects of Mutations on the UPF1 Protein Stability

**Figure 2:**
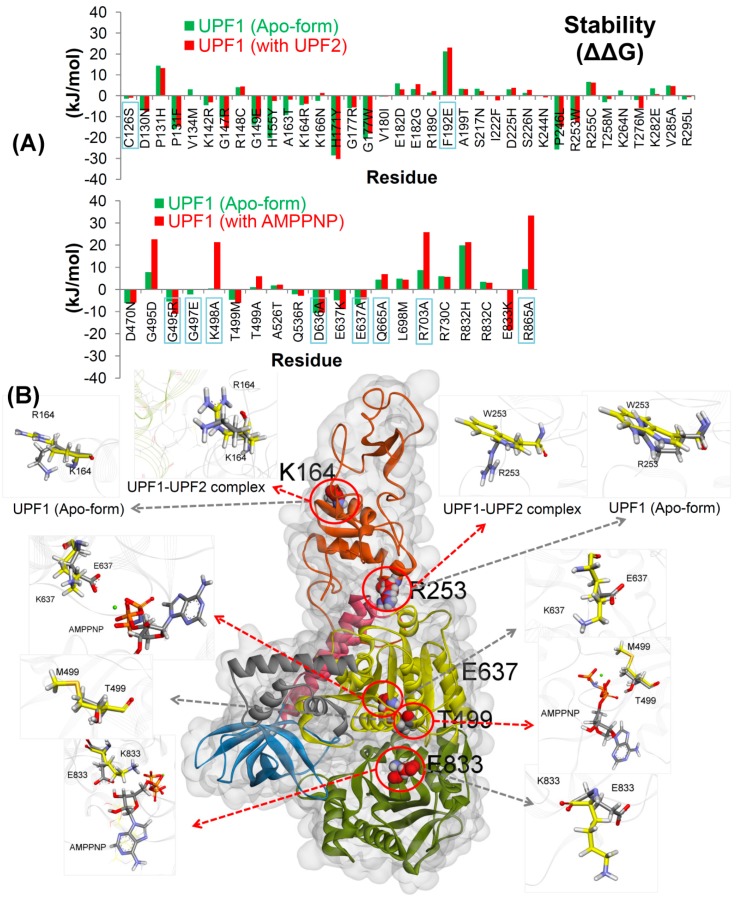
Effects of mutations on the UPF1 protein. (**A**) UPF1 structural stability change (∆∆G; kJ/mol) upon mutation. In addition, the mutations from previous studies [4,7,11,13,19,21,32,33] are indicated by labels with the blue box. (**B**) The UPF1 structure showing mutations studied by MDS. The red arrows represent UPF1 mutations in the complex with UPF2/AMPPNP and the grey arrows show mutations in the apo-form of UPF1. Cα atoms of the wild-type and mutated residues are shown in grey and yellow, respectively (different domains of UPF1 are colored as per Figure 1). Color scheme: oxygen in red, hydrogen in silver, nitrogen in blue, and sulfur in orange.

The recurrent cancer mutations analysis in the UPF1 gene [29] using the cancer genome atlas (TCGA) sequencing data shows that 31 residues located in the UPF2 interacting domain (i.e., CH-domain) of UPF1 and 10 residues at the ATP-binding site/substrate pocket were found to be frequently mutated (Figure 1 and Figure S2). The UPF1 residues interacting with AMPPNP as well as residues around 8 Å of AMPPNP were characterized and mutation association with different cancer types was considered (Figure S2). Moreover, mutations retrieved from previous studies (C126S and F192E [4,7,11] in the CH-domain, and in the nucleotide-binding site: G495R, G497E, K498A, D636A, E637A, Q665A, R703A, and R865A [19,21,32,33]) were also subjected to our stability change calculations (i.e., for the UPF1 protein), in order to perform comparative analysis with the cancer mutations from cBioPortal [29]. In line with these observations, all mutations were generated and screened for changes in structural stability upon point mutations in UPF1 with respect to the wild-type structure. The analysis was performed for UPF1 apo-form and for the UPF1 mutant models in complex with UPF2 or AMPPNP (Figure 2A). The mutations showing more negative (∆∆G) values compared to other variants, are those that result in improving the stability of the UPF1 protein (Figure 2).

Findings from the stability change suggest that the mutations C126S and F192E (from the CH-domain) destabilize the UPF1 protein (Figure 2A), and this correlates with the previous studies in which the C126S mutation abolishes the interactions between UPF1 and UPF2 [4,7], and the F192E mutation disrupts the hydrophobic pocket in the CH-domain and interferes with allosteric regulation [11,21].

In addition, previous studies of both yeast and human UPF1 have shown that the mutants K498A, D636A, and E637A were strongly defective for ATPase activity, but only K498A was defective in ATP-binding [13,19,33]. As shown in Figure S2C, the residue K498 is involved in interactions with AMPPNP (tri-phosphate moiety) and the K498A mutant was not able to form any interaction with the AMPPNP molecule. This is consistent with the stability change analysis indicating that the K498A mutant destabilizes the UPF1 protein (Figure 2A). It is also known that the G495R/G497E mutants in the substrate pocket prevent dephosphorylation [32]; in our analysis, mutant G497E was found to be slightly destabilizing (Figure 2A). Additionally, the UPF1 mutants R703A and R865A are defective in both ATP-binding and hydrolysis, whereas the Q665A mutant nearly eliminated the ATPase activity [19]. In our findings for the wild-type system (Figure S2C), these residues were involved in the interactions with the tri-phosphate moiety of AMPPNP, and their mutations Q665A, R703A, and R865A destabilize the UPF1 protein and hinder their interactions with AMPPNP (Figure 2A).

The individual variants resulting in significant structural changes and improvement in (i) the UPF1 protein stability, (ii) located in the binding interfaces of UPF1 with UPF2 and (iii) placed at the UPF1 binding interface to AMPPNP were selected for further detailed analysis by MDS (to identify molecular properties). In the CH-domain, theK164R and R253W alleles located at the UPF1-UPF2 interface were analyzed by MDS. The K164R mutation is located at the UPF1-UPF2 interface while the R253W mutation is located at the boundary between the CH-domain and the helicase regions. As shown in Figure 2, UPF1 (CH-domain) residue R253 promotes its conformation between its apo-state and when in complex with UPF2. Therefore, MDS was used to explore whether this acts as a switch between the intramolecular interactions between the CH-domain and the helicase regions.

Moreover, for the selection of mutations in the ATP/AMPPNP-binding region to be analyzed by MDS, a criteria was set to identify those mutations that improved the stability of the active form and destabilized the inactive form (apo-form) of the UPF1 protein, as well as those mutations located close to the phosphate groups of the AMPPNP molecule in the substrate binding pocket. We identified three mutants T499M, E637K, and E833K, of which the mutant E833K stabilizes UPF1 in complex with AMPPNP but not in its apo-form (Figure 2). The coupling of conformational changes to the nucleoside triphosphate, usually ATP-binding and hydrolysis is a general mechanism by which helicases and motor proteins catalyze physical transitions [19]. In conclusion, MD simulations can identify the conformational changes in UPF1 mutated at the ATP-binding site/substrate pocket (T499M, E637K, and E833K) and mutant at the interface with UPF2 (K164R and R253W).

### 2.2. Molecular Dynamics Simulations

The wild-type UPF1 protein was simulated in the apo-form as well as in the complex with UPF2 or the non-hydrolyzable ATP analogue (AMPPNP). Five single mutations were studied in the inactive apo-form of UPF1 and in the complex with UPF2 (K164R and R253W), or with AMPPNP (T499M, E637K, and E833K).

#### 2.2.1. UPF2 and AMPPNP Stabilizes the Wild-Type UPF1

**Figure 3:**
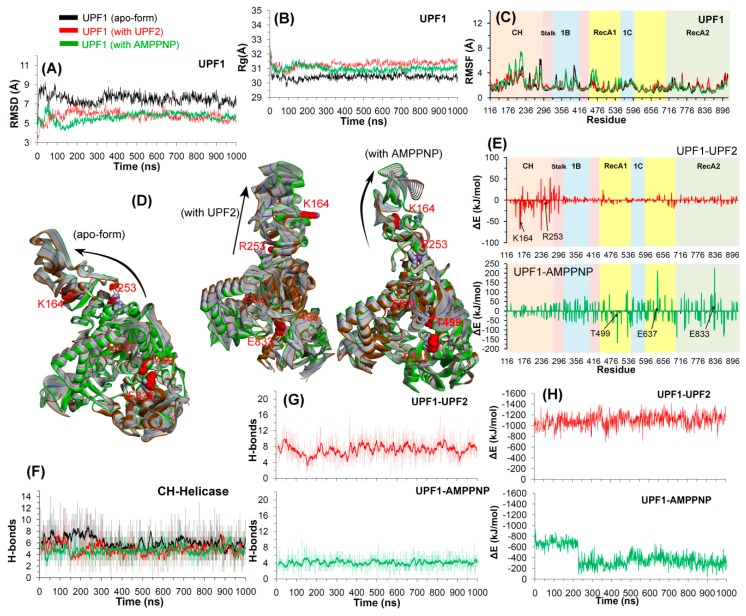
Structural analysis of wild-type UPF1 in the MD simulations. (**A**–**C**) RMSD, radius of gyration profile, and RMSF representing the structural changes and fluctuations of residues inthe UPF1 protein. (**D**) The protein motion corresponding to the first eigenvector defined on the basis of the combined trajectories (green is from the beginning, blue from 400 ns (where the system stabilized), and brown from the end of the MD). (**E**,**H**) Energy contribution of each UPF1 residue to the binding with UPF2/AMPPNP and the total binding energy for UPF1-UPF2/AMPPNP, calculated using MM-PBSA. Contribution of residues selected for mutation analysis are labelled and shown with black arrows in (**E**). (**F**,**G**) Number of hydrogen bonds formed between the CH-helicase domains (intramolecular UPF1) and between UPF1-UPF2/AMPPNP (intermolecular). The dark lines represent the moving average of H-bonds formed with a period of 10 ns (i.e., number of H-bonds averaged every 10 ns).

The time dependent changes in the root mean square deviation (RMSD) of the non-hydrogen atoms and the radius of gyrations (Figure 3A,B) suggest that the simulated systems attained stability around 400 ns. For the apo-form and UPF1-UPF2 or UPF1-AMPPNP simulations, the average RMSD values were between 8 and 9 Å and 6 and 7 Å, respectively (Figure 3). We next examined the root mean squared fluctuations (RMSF), a metric of residue flexibility, to measure the effects of UPF2 and AMPPNP binding (Figure 3C). All three wild-type systems (apo-form, UPF1-UPF2, and UPF1-AMPPNP) showed similar distributions (Figure 3C); high fluctuations were found in the CH-domain (the 116–295 region) of UPF1 that connects to the UPF2 protein (Figure 3C).

The radius of gyration (R_g_) is an indicator of the protein secondary structure compactness. R_g_ measures the mass of atoms relative to the center of the mass of a protein or molecule; higher values of gyration indicate less compactness. The wild-type structure of UPF1 was found to be less compact in complex with UPF2 or AMPPNP compared to that of the apo-state (Figure 3B). Presentation of potential motions projected along the first eigenvector or the principal component showed that the absence of any interacting partner (UPF2 or AMPPNP) drives the movement of the UPF1 CH-domain towards the helicase-domain as shown in Figure 3D. Moreover, the number of hydrogen bond interactions was generally constant for all systems, implying successful equilibration of the CH-helicase domain interactions as well as the UPF1-UPF2 interactions (Figure 3F,G). In the apo-state of the UPF1 protein, the CH- and helicase-domains engage in a relatively higher number of H-bonds and were found to be reduced in complex with UPF2 or AMPPNP. This observation correlates with a previous study suggesting that binding of UPF2 with the UPF1 CH-domain, disrupts the interactions of the CH-domain with the catalytic helicase-domain, which then promotes the UPF1 helicase activity [11]. The interactions of UPF1 with UPF2 and with AMPPNP were stabilized throughout the simulation by about eight and five hydrogen bonds, respectively (Figure 3G).

From the equilibrated trajectories of UPF1-UPF2 or UPF1-AMPPNP simulated systems, the binding free energy (∆G_bind_) was computed for the snapshots (structures) collected every 1 ns, using the molecular mechanics/Poisson–Boltzmann surface area (MM-PBSA) method. The findings obtained from this approach can help to better understand the interaction pattern of UPF1 with UPF2 or AMPPNP, as well as unveil the hot spot residues of UPF1 that were common in the wild-type and mutant systems. The averaged ∆G_bind_ for the UPF1-UPF2 complex was −1122 kJ/mol (Figure 3A) and for the UPF1-AMPPNP complex was −339 kJ/mol (Figure 3H), considering the stabilized systems. The residue level contributions in binding energy suggest that the residues 116–296 of UPF1 (CH-domain) make significant contributions to the interactions with UPF2 (Figure 3E) and the residues within the range 438–896 in UPF1 (helicase-domain; RecA1 and RecA2) showed significant contributions to the interactions with AMPPNP. Moreover, these results are consistent with previous studies [11], demonstrating that the CH-domain contributes to UPF2 binding, and the RecA1 and RecA2 domains contribute to substrate binding.

Furthermore, the intramolecular H-bonds (within UPF1) formed between the CH- and helicase-domains were analyzed (Table S2), and the H-bond occupancy was defined as the percentage of the total simulation time a specific H-bond existed. The findings suggest that two interacting pairs of residues (CH/helicase) were found to be common in the apo-form and in the UPF1-UPF2 complex: R253/Y300 and R253/V437, respectively. In addition, the common interacting pair of residues in the apo-form and in the UPF1-AMPPNP complex was R253/A431. Residues R253 and E278 of the CH-domain, and Tyr300 of the helicase-domain of UPF1 were found to be engaged in intramolecular H-bond interactions in all three simulated systems. Residues of the CH- or helicase-domain making a long-lasting interaction network are presented in the Table S2. As shown in Table S3, most of the intermolecular interactions (UPF1-UPF2 or UPF1-AMPPNP) are characterized by high H-bond occupancy which suggests stable as well as conserved interactions. The UPF1 residues S152, S203, and V205 formed stable H-bonds with UPF2 with occupancy ≥40%, and residues G495, K498, and T499 were engaged in H-bonds with the AMPPNP molecule having occupancy ≥50% (Table S3).

#### 2.2.2. Effects of Cancer Variants in the ATP-Binding Pocket of UPF1

**Figure 4:**
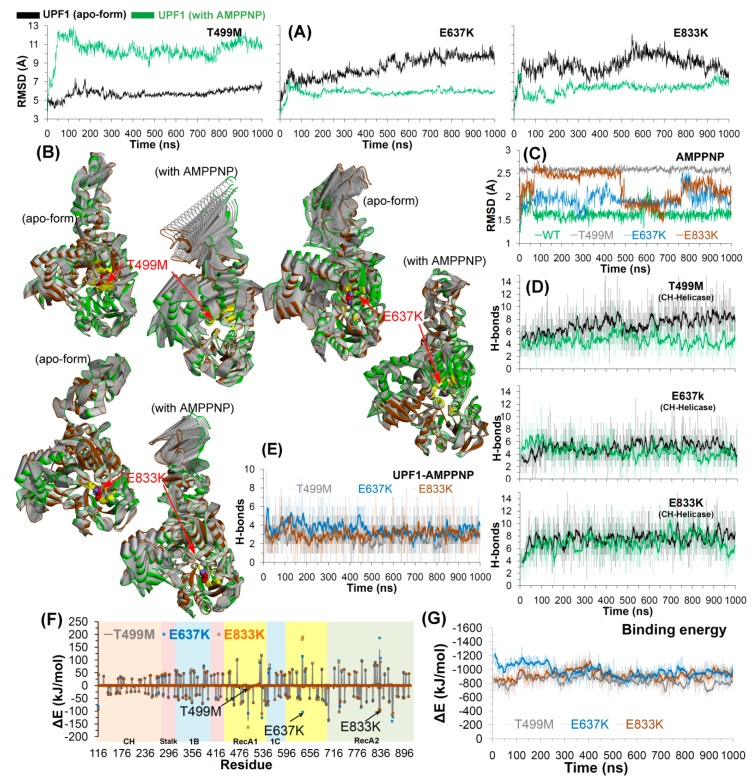
Structural analysis of T499M, E637K, and E833K mutants in the UPF1 protein. (**A**) RMSD for all atoms (excluding hydrogens) of mutant UPF1. (**B**) The principal motion projected along the first eigenvector defined on the basis of the combined trajectories (green marks the beginning and brown is the end of MDS; yellow are residues within 5 Å of the mutated residues). (**C**) RMSD for all atoms (excluding hydrogens) of AMPPNP from the WT and mutant UPF1-AMPPNP systems. (**D**,**E**) Number of hydrogen bonds formed between CH-helicase domains (UPF1 intramolecular) and between UPF1-AMPPNP (intermolecular). The dark lines represent trends with a moving average of H-bonds formed with a period of 10 ns (i.e., number of H-bonds averaged every 10 ns). (**F**,**G**) Energy contribution (computed using MM-PBSA) of each residue of UPF1 to the binding with AMPPNP and total binding energy for UPF1-AMPPNP complex, respectively. Contribution of residues selected for mutational analysis are labelled and shown with arrows in (**F**).

Three single amino acid mutations (T499M, E637K, and E833K) in the ATP-binding site/substrate pocket of UPF1 were studied by the MDS approach. All three mutants showed different deviations in the apo-state and when bound with AMPPNP. For the UPF1 mutants E637K/E833K, RMSDs were higher for the apo-form with sudden spikes, whereas the RMSD remained relatively stable for UPF1 in complex with AMPPNP. A contrasting behavior was observed in the case of the mutant T499M, with RMSD values higher by about 5 Å for the UPF1-AMPPNP complex compared to the apo-form (Figure 4A). The PCA data suggests greater movements of the CH-domain in the UPF1 mutants T499M and E833K when complexed with AMPPNP, whereas this domain was relatively stable in the apo-state. In the case of the E637K mutant, the structure of UPF1 has increased motions in the apo-form compared to that in complex with AMPPNP (Figure 4B).

Moreover, for the AMPPNP molecule, sudden spikes in RMSDs were observed when complexed with the UPF1 mutant systems E637K or E833K, and these jumps arise from the rotations of the aromatic rings of AMPPNP or its movement within the UPF1 substrate pocket. In addition, for the mutant T499M system, the RMSD of AMPPNP reached ~2.5 Å and then remained stable throughout the MD simulation (Figure 4C).

For all UPF1 substrate pocket mutant systems (T499M, E637K, and E833K), the average number of H-bonds formed between the CH- and helicase-domains was in the range of ~4–8 (Figure 4D), and the residues making long-lasting interactions were also identified (Table S4). The higher number of residues were identified as engaging in high occupancy intramolecular (between CH-helicase domains) H-bond interactions in the apo-form of mutant UPF1 (T499M/E833K) compared to the mutant systems bound with AMPPNP (Table S4). In addition, the average number of intermolecular H-bonds formed between AMPPNP and mutant UPF1 (T499M/E637K/E833K) was around three (Figure 4E). However, in the mutant T499M system, a slightly lower number of H-bonds (average ~2.7) were formed with the AMPPNP molecule (Figure 4E).

Using the MM-PBSA approach, the binding energy between AMPPNP and UPF1 substrate pocket mutants were estimated (the entropic contributions in the binding energy were not included; Figure 4F,G). The ability of UPF1 to interact with the ATP analogue was found to be similar in the E637K and E833K mutant systems, with ∆G_bind_ average of around −900 to −1000 kJ/mol, whereas a slightly lower ∆G_bind_ (average: −833 kJ/mol) was observed for UPF1-AMPPNP in the T499M mutant system (Figure 4G). In addition, to obtain a more-detailed insight into the binding of AMPPNP to the three UPF1 mutant systems, the ∆G_bind_ was further decomposed into individual residue contributions. As shown in Figure 4F, UPF1 residues contributing significantly to binding AMPPNP belonged to the RecA1 and RecA2 regions of UPF1 in all systems.

**Figure 5:**
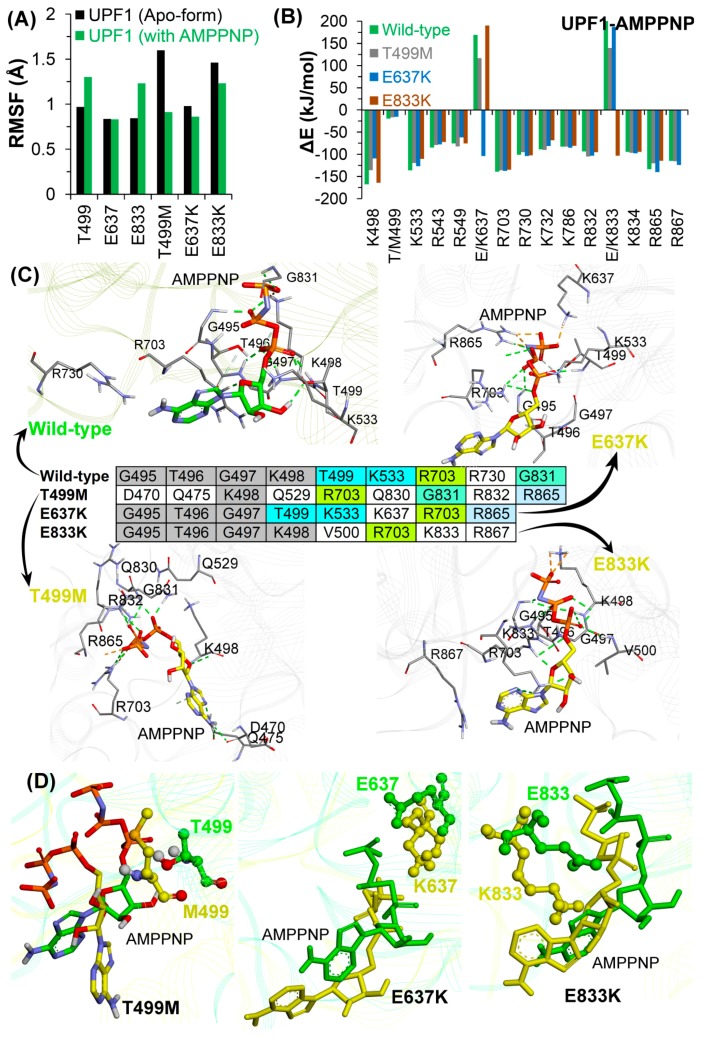
Molecular properties of the UPF1-AMPPNP model systems. (**A**) Substrate binding site residues of the UPF1 protein and studied mutations. RMSFs for wild-type UPF1, mutant models; apo-form and AMPPNP bound UPF1. (**B**) Individual residues of UPF1 contributing to the binding energy for AMPPNP, calculated by MM-PBSA. (**C**) Binding mode of AMPPNP to the UPF1 protein in different model systems, and UPF1 residues forming stable H-bonds with AMPPNP having occupancy ≥10%. (**D**) Binding conformation of AMPPNP with respect to the wild-type and mutated UPF1 (T499M, E637K, and E833K) obtained from the end of MDS.

The radius of gyration (Figure S3) ranged between ~29–32 Å for the UPF1 substrate mutant systems, and overall the RMSFs exhibited similar trends in conformational flexibilities, ranging between 2–4 Å. The CH-domain (residues 116–236) of UPF1 had relatively higher RMSFs of up to 8 Å in all systems, and this higher flexibility is likely due to the absence of UPF2 in the system (Figure S3). RMSFs for each mutated residue were compared with their behavior in wild-type UPF1 in the apo-state and bound to AMPPNP (Figure 5A). The residues T499 and E833 in the wild-type UPF1 protein showed slightly higher fluctuations when bound with AMPPNP compared to the apo-form, whereas these residues in the UPF1 mutant showed higher RMSFs when in the apo-form compared to the AMPPNP bound systems. In addition, the RMSF ofE637 was ~1 Å in the apo-state and AMPPNP bound forms in both wild-type and mutant UPF1 (Figure 5A).

The UPF1 residues contributing ≤−80 kJ/mol (∆G_bind_) to the total binding energy with AMPPNP were identified, and their behavior was compared with other simulated models. Almost all of the residues in the mutant protein exhibited similar contributions to binding AMPPNP as those of the wild-type UPF1, except E637 and E833 which contributed more in their mutant forms E637K and E833K, respectively, compared to their native form (Figure 5B).

Interactions and the binding pattern analysis of AMPPNP showed that the tri-phosphate moiety of the AMPPNP molecule (Figure 5C) engages with a range of residues in the wild-type and mutant systems. Particularly, the residue R703 of the UPF1 protein formed hydrogen bond interactions with the AMPPNP in the wild-type as well as in all mutant systems (T499M, E637K, and E833K). Residues of UPF1 that formed H-bond interactions with AMPPNP in the wild-type and in the E637K and E833K mutant models were: G495, T496, and G497 (Figure 5C). In addition, the residue K498 was found to be engaged in H-bonds with the AMPPNP in the wild-type, T499K, and E833K systems, whereas the residues T499 and K533 were involved in H-bond interactions with AMPPNP in the wild-type and E637K systems (Figure 5C). Residue G831 formed H-bonds with AMPPNP in the wild-type as well as in T499M models, and R865 formed H-bonds with AMPPNP in T499M and E637K mutant systems (Figure 5C and Table S5). Analyzing the interactions of mutated residues (T499M, E637K, and E833K) with AMPPNP (Figure 5C) identified interactions with residues K637 and K833 but not with the M499 mutant.

Findings from MDS suggest that the wild-type and mutant UPF1-AMPPNP complexes showed significant differences in the conformations of the AMPPNP molecule in the substrate binding pocket of UPF1, particularly, in the mutant T499M (Figure 5D). By contrast, the other two mutant model systems E637K and E833K exhibited slight movements observed for AMPPNP, but lacked any significant conformational shift as that seen for the T499M system (Figure 5D). To understand this difference in the AMPPNP conformation, we traced the dynamics of the mutated residues in the apo-form and in the UPF1-AMPPNP bound form (Figure S4). Analyzing the wild-type systems, the dynamics of all three residues T499, E637, and E833 showed almost similar conformations in the apo-form and AMPPNP bound forms (Figure S4). In addition, for the E637K or E833K mutant model systems, the conformations of the mutated residues K637 or K833 in the apo-state correlated with the AMPPNP-bound systems. By contrast, the mutated residue M499 showed different conformations in the apo-form and AMPPNP-bound form (Figure S4). The dynamics of AMPPNP and the mutated T499M residue suggest that after mutation the residue M499 reduces the stabilization of the tri-phosphate moiety of the AMPPNP in the substrate binding site of UPF1 (Figure 5D). This suggests that the T499M mutation may also affect the ATP-binding/hydrolysis process of the UPF1 protein or the NMD machinery.

#### 2.2.3. CH-Domain Mutants Enhance UPF1-UPF2 Binding

**Figure 6:**
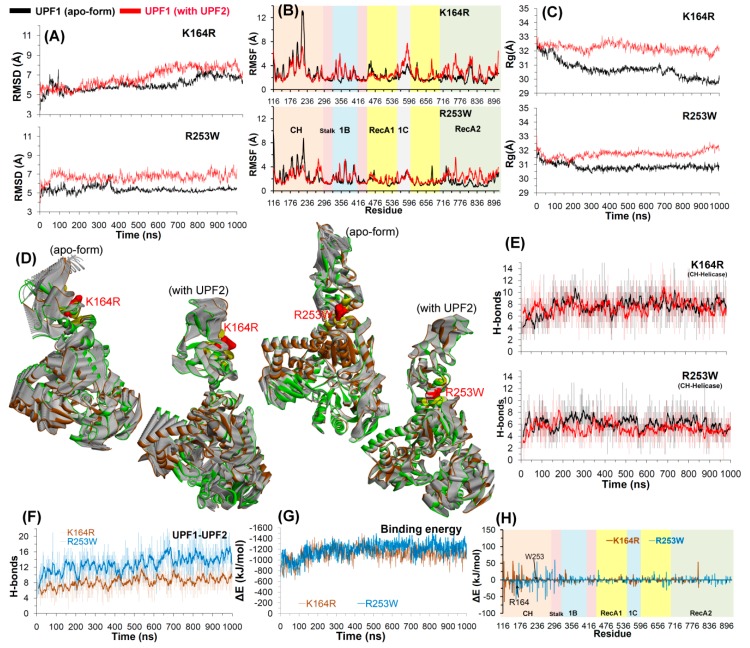
Structural analysis of UPF1 mutants K164R and R253W. (**A**–**C**) RMSD, RMSF, and radius of gyration profiles representing structural changes and fluctuations of UPF1 residues. (**D**) The motion corresponding to the first eigenvector defined on the basis of the combined trajectories. (**E**,**F**) Number of hydrogen bonds formed between CH-helicase domains (UPF1 intramolecular interactions) and between UPF1-UPF2 (intermolecular interactions). The dark lines represent trend with a moving average of H-bonds formed with a period of 10 ns (i.e., number of H-bonds averaged every 10 ns). (**G**,**H**) Energy of each residue (of the mutant UPF1) contributing to the binding with UPF2 and total binding energy for UPF1-UPF2, calculated by MM-PBSA.

MDS was used to investigate how mutations in the UPF1 CH-domain affect its binding and interactions with UPF2. The RMSD for both UPF1 mutants in the apo-form as well as in complex with UPF2 were found to vary between ~5 and 7 Å. In case of the mutant R253W, the RMSD remained relatively stable during the last 500 ns, whereas for the UPF1 with mutation K164R, the stable behavior was observed after ~600 ns (Figure 6A). Similarly, the K164R mutant had similar RMSD statistics for the apo-form and UPF1-UPF2 complex. In addition, for the R253W simulation, RMSD was higher by ~2 Å for UPF1 in complex with UPF2 compared to the apo-state (Figure 6).

**Figure 7:**
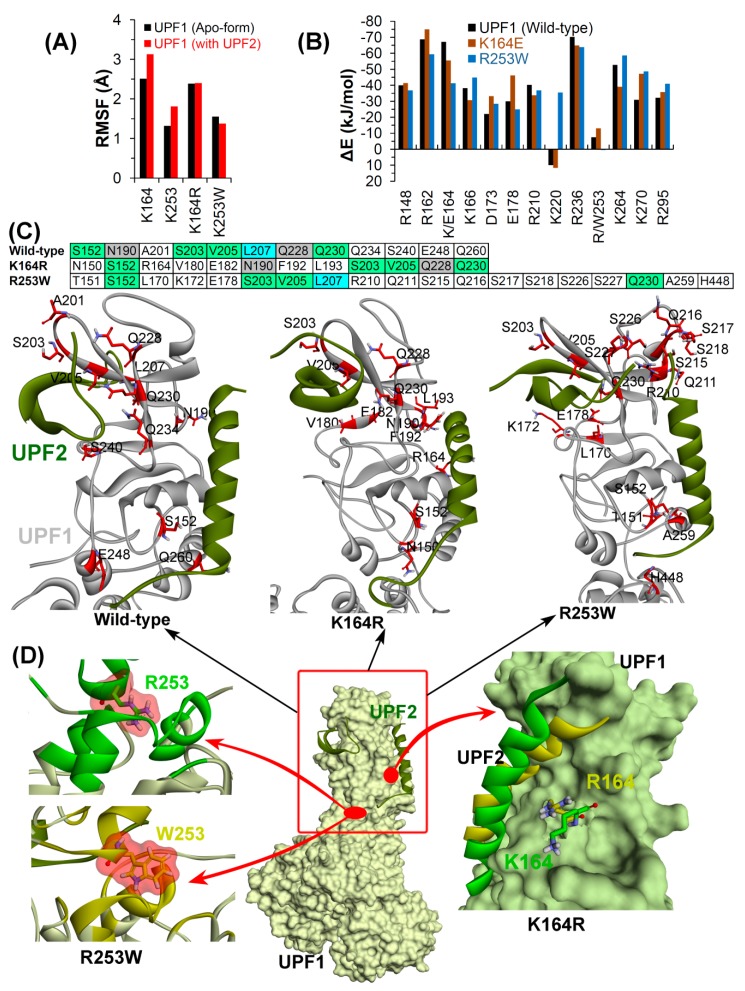
Detailed analysis of UPF1-UPF2 model systems. (**A**) UPF1 residues involved in binding with UPF2 and their mutations. RMSF values were calculated in native or wild-type UPF1, mutant models, apo-form, and UPF1 bound with UPF2. (**B**) Individual UPF1 residues contributing to the binding energy with UPF2, were computed using MM-PBSA. (**C**) Interactions of UPF1 with UPF2 in different model systems, and residues of UPF1 forming stable H-bonds with UPF2 having occupancy ≥10%. (**D**) Binding mode or conformation of the UPF1 residues (K164 and R253 in native or mutated form R164 and W253) with UPF2 (Color scheme: green represents wild-type and yellow mutated systems, carbon in green/yellow, oxygen in red, nitrogen in blue, and hydrogen in silver).

RMSF for each residue in UPF1 showed that the residues ranging from 116–295 of the CH-domain fluctuate more than any other region of the protein, and peaks represent the local fluctuations along the protein (Figure 6B). In the mutant UPF1-UPF2 systems, particularly in R253W, residues in the range 210–230 (from CH-domain) showed less fluctuations compared to the wild-type, as this region makes interactions with the UPF2 protein (Figure 6B and Figure 7). For the UPF1(K164R)-UPF2 system, some regions in the helicase-domain show high fluctuations compared to the UPF1(R253W)-UPF2 system; this can be due to lack of CH-helicase (intramolecular) interactions in the K164R system that were observed in the R253W system (Figure 7). Moreover, UPF2 interacts with the mutated K164R residue and to achieve this interaction, the UPF2 protein adopts a slightly different (bent) conformation which resulted in the loss of some interactions with UPF1 in the vicinity of the CH-helicase domain (Figure 6B and Figure 7).

The radius of gyration was computed for mutant models to observe the conformational alterations of the K164R and R253W mutants in their apo and UPF2 bound forms. The structure of mutant UPF1 (K164R/R253W) in its apo-form exhibited lower Rg values compared to that with UPF2, suggesting that in its apo-state the mutant is more compact (Figure 6C).

In the eigenvalue projections (PCA), the clusters were more well defined for UPF1 mutants complexed with UPF2, whereas in the apo-form, regions of mutant UPF1 close to the CH-domain had larger movements (Figure 6D). The analysis of H-bond interactions formed between the CH- and helicase-domains of UPF1 shows that the number of these intramolecular interactions were conserved with ~4–8 hydrogen bonds in all simulated systems (Figure 6E). The common residues of CH-domain interacting with helicase-domains in the mutant apostate (K164R/R253W) were: R253/W253, R255, T258, Q261, and L293. In addition, for the mutant (K164R/R253W) UPF1-UPF2 complexes, residues T258, Q260, Q261, and D279 of the CH-domain were found to be engaged in intramolecular H-bonds. In the helicase-domain, residues that formed H-bonds with the CH-domain in the mutant (K164R/R253W) apo-state were: Y296, E297, and D298. Residues E297, D298, and Y300 of the helicase-domain formed H-bonds with the CH-domain in both mutant UPF1(K164R/R253W)-UPF2 complexes (Table S6).

The average number of H-bonds formed between mutant UPF1-UPF2 was in the range of ~8–10 for K164R and ~14–16 for R253W systems, and these numbers were maintained throughout MDS (Figure 6F). The total binding energy (contribution from the van der Waals, electrostatic, polar, and non-polar interactions) for UPF1-UPF2 was found to be almost similar, −1200 to −1400 ∆E (kJ/mol) in the case of both mutant models, K164R and R253W (Figure 6G). Moreover, a quantitative assessment of the binding energy at individual residue level shows that in both UPF1(K164R/R253W)-UPF2 systems, the residues with high contributions are placed in the CH-domain (residues ranged 116–296; Figure 6H).

Four intermolecular (UPF1-UPF2) H-bond interactions were found to be common in both complexes of UPF1(K164R/R253W)-UPF2 (Table S7), and these common H-bonds suggest stable as well as conserved interactions. The interacting pairs of residues UPF1-UPF2 were as follows: S152-D1110, S203-V1172, V205-V1172, and V205-L1174, respectively. Moreover, the individual residues Q230 of UPF1 as well as N1124 and Q1127 of UPF2 were found forming common intermolecular H-bond interactions in both K164R or R253W systems. Globally, the UPF1(R253W)-UPF2 interactions were more stable with relatively high H-bond occupancy compared to the UPF1(K164R)-UPF2 interactions (Table S7).

RMSFs for the specific UPF1 residues (K164 and K253) and their structural fluctuations in mutated systems were compared with the wild-type systems in both apo as well as UPF1-UPF2 complexes. The findings suggest that both residues K164 and K253 of wild-type UPF1 showed slightly higher fluctuations in complex with UPF2 compared to the apo-form, whereas these residues undergo similar fluctuations in mutant models whether UPF1 was in the apo-state or was UPF2 bound (Figure 7A). Our findings suggest that UPF1 residues R148, R210, R236, and R295 contributed similar binding energies to UPF2 in wild-type as well as in mutated systems (Figure 7B).

The binding mode of UPF1-UPF2 proteins and residues of CH-domain forming stable (occupancy ≥10%) H-bonds with UPF2 in different model systems are highlighted in Figure 7C. Interacting (H-bond) residues common to all three UPF1-UPF2 complexes were S152, S203, S205, and Q230. Furthermore, residues N190 and Q228 of UPF1 interacting with UPF2 were found common in wild-type and K164R systems. In addition, the L207 residue of UPF1 formed interactions with UPF2 in the wild-type and R253W systems (Figure 7C). The binding mode of the UPF1 with UPF2 protein as well as the conformation of the residues K164 and R253 binding in their native and mutated forms (R164 and W253) are shown in Figure 7D.

Figure S5 represents RMSDs of the UPF2 protein from different simulated systems, and it was observed that the RMSD of UPF2 remained almost stable after ~500 ns in all three complexes (wild-type/K164R/R253W). The RMSD of the UPF2 protein in complex with mutant UPF1(K164R/R253W) was higher by ~1–2 Å compared to its complex with the wild-type UPF1. In addition, a quantitative assessment of the binding energy of individual residues was also performed for the UPF2 protein, and the residues of UPF2 with high contributions to binding UPF1 wild-type/mutant models were also identified (Figure S5).

#### 2.2.4. Conformations of the Wild-Type and Mutant Systems

**Figure 8:**
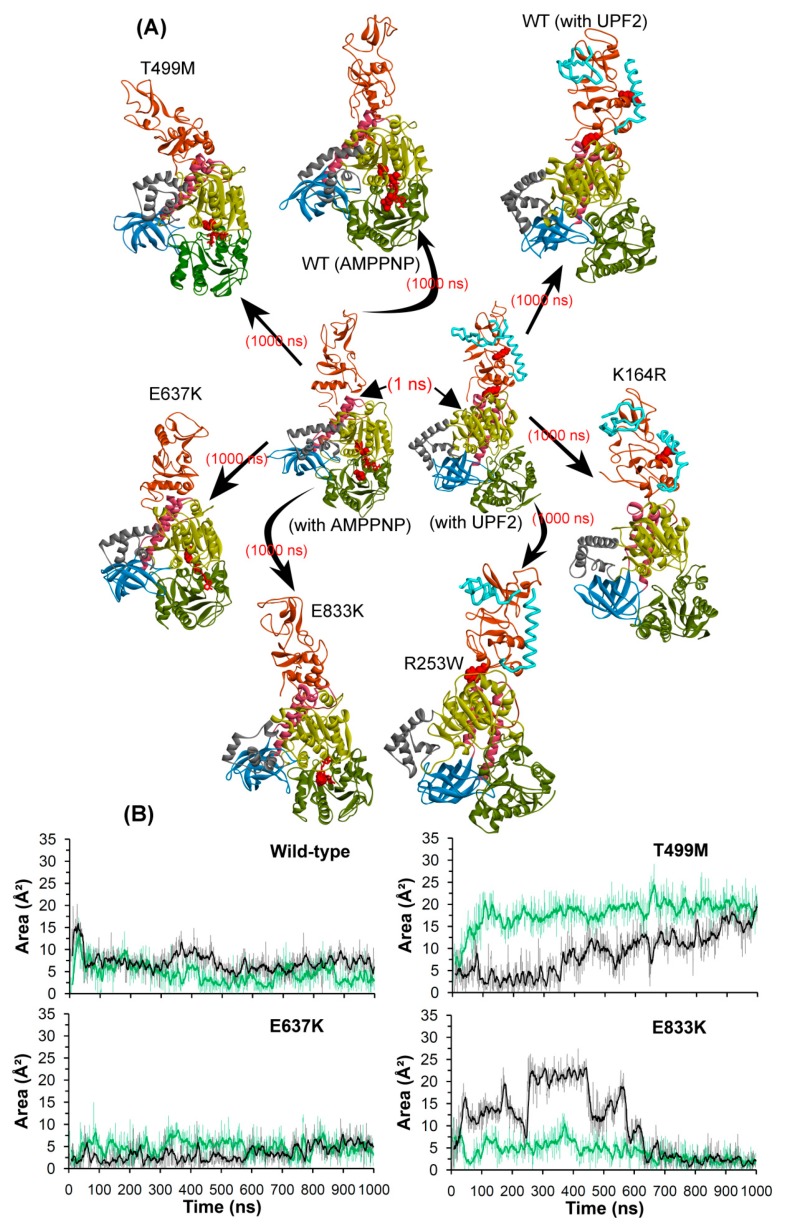
Structural/conformational changes of UPF1 protein when complexed with UPF2 or AMPPNP. (**A**) Wild-type and mutant UPF1 protein in complex with AMPPNP or UPF2. (**B**) Area analysis of structural changes in the ATP-binding region of the UPF1 protein, triangle selected for area calculation was based on Cα atoms coordinates of T499, E637, and E833 residues. The dark lines represent the trend with a moving average of area with a period of 10 ns. Different domains of UPF1 are colored as per Figure 1.

The structural transitions of the UPF1 protein were analyzed from the beginning and end of the MD simulations. There is considerable conformational flexibility observed in some regions of UPF1, mostly the CH-domain situated at the interface interacting with UPF2 and in the domains binding/close to AMPPNP (Figure 8). For the systems with UPF2, the CH-domain of UPF1 maintained its native structure which suggests that the intermolecular interactions of UPF1-UPF2 are required for maintaining the native state conformation. Moreover, concerning the UPF1-AMPPNP complexes, the CH-domain does not undergo much movement in its wild-type form, whereas in all three substrate pocket mutant models, this region is mobile in the direction of the helicase-domain (Figure 8).

Comparing the dynamics of UPF1 substrate pocket mutants: T499M, E637K, and E833K it is apparent that the CH-domain undergoes more conformational changes in its AMPPNP bound form when compared to its apo-form (Figure S6). Moreover, the subdomain rearrangements in the UPF1 substrate pocket mutant models describe that the CH- and helicase-domains develop their spatial proximity in the presence of AMPPNP, and such behavior was not seen in any of the other simulated systems (Figure 8 and Figure S6). This analysis suggests that motions of the CH-domain are directly affected by the mutations in the substrate pocket of UPF1, and also hint at the complex nature of allosteric transitions. For example, it is possible that the substrate binding site mutations enable the CH-domain to move towards the helicase region such that the AMPPNP molecule would be stuck in the ATP-binding site, which can affect or stop the hydrolysis reaction.

The compactness or flexibility of the substrate pocket were analyzed by calculating an area encompassed by selecting the Cα atoms (xyz coordinates) of a set of three residues T499, E637, and E833. The area computed can serve as a measure of the open and closed spaces accessible to the ATP ligand or its analogues (e.g. AMPPNP; Figure 8B). For the wild-type and mutant E637K systems, the area computed between the three residues was in the range of around 5–10 Å^2^, whereas for the T499M and E833K mutant systems frequent jumps were observed in these values. Particularly, in case of the T499M system the area increased significantly, reaching up to ~20 Å^2^ in the apo state and ~25 Å^2^ in the AMPPNP bound system by the end of MDS. During the initial part of the simulation of the E833K system, the area occupied by the substrate binding region in the UPF1 protein was considerably different for the apo-form and AMPPNP bound form, but later (600–1000 ns), this region became more compact in both systems (Figure 8).

Furthermore, the area accessible by the three residues T499, E637, and E833 (of the ATP-site) in the UPF1 protein when complexed with UPF2 differs between the apo-form and UPF1-UPF2 systems as shown in Figure S7. In apo systems (wild-type/K164R/R253), this area was around 10 Å^2^, and for the wild-type UPF1-UPF2 complex the area ranged between 40 and 60 Å^2^. Concerning the UPF1(K164R)-UPF2 complex, the area occupied was about 60 Å^2^ initially, but eventually converged to ~30 Å^2^. In addition, the UPF1(R253W)-UPF2 system had the highest area (~90 Å^2^). These variations in area reflect the flexible nature of the ATP-binding region in the UPF1 protein, and this underscores how this region can undergo induced fit in order to accommodate the AMPPNP molecule (Figure S7 and Figure 8).

## 3. Discussion

The UPF1 protein is a master regulator of NMD machinery, and the ability of NMD to selectively target PTC-containing mRNAs depends on the ATPase and helicase activities of UPF1. In addition, the UPF1 and UPF2 proteins contribute/constitute the core of the NMD pathway. NMD can have impacts on health; for example, the accumulation of mutated mRNA can lead to enhanced production of mutant proteins that can result in increased disease incidence [34]. In addition, the inhibition of NMD can allow the re-expression of mutant proteins in cancer models and can stimulate tumor death [35]. These latter data suggest that the NMD protein components could form a therapeutic target in selected human cancers. Indeed, the inhibition of NMD in mouse models can cause anti-tumor immunity through the production of tumor-specific T-cells that target neo-antigens on the surface of cancer cells [36]. However, targeting NMD could be complicated by whether or not NMD pathway components are mutated in human cancers. In this study, we set out to first determine whether the NMD pathway component UPF1 was in fact mutated to a degree in human cancers; and if so, what the effects of these mutations might be on the protein function. For example, mutations could be loss or gain-of-function, depending upon the reaction cycle and mode of action of each regulatory domain. This information is important to facilitate drug-discovery screens aimed at inhibiting tumor cell growth or stimulating neoantigens that can promote anti-tumor immunity.

Using the large annotated multi-tumor cancer genome sequencing resource, we identified 41 mutations (31 residues in the CH-domain and 10 residues at the ATP-binding site) across the coding region of the core component of the NMD pathway, UPF1 (Figure 1C, Figure 2, and Figure S2). As an example, ovarian cystadenocarcinoma, the missense mutation rate was relatively low, but a relatively high rate of gene amplification was observed (Figure S8), suggesting that the UPF1 activity might be amplified in this particular cancer type. However, any statistically significant co-amplification of UPF1 or the UPF1 binding protein UPF2 (Figure S8) was lacking. This suggests that any one of these two components of the NMD pathway might be rate-limiting, and the amplification of UPF1 or UPF2 might be sufficient to stimulate the core UPF1 function. Thus, this ovarian cancer type might be an attractive target for UPF1 drug leads, under conditions in which patients have an UPF1 or UPF2 amplification that stimulates the UPF1-dependent NMD pathway.

Analysis of other cancer types using TCGA has shown a similar selectivity to ovarian cancer; in lung cancer or esophageal cancer, the total amplification rate was lower than that for ovarian cancer, but the missense mutation rate was higher in either UPF1 or UPF2 (Figure S8). However, these missense mutation cases lack confident data as to whether the mutations are loss or gain-of-function. This complicates the utility of using genetic mutation of UPF components from cancer genome sequencing as an argument for stratifying patients for trials with UPF1 drug leads. As such, we focused this current study on understanding the effects of missense mutations in the core NMD component and UPF1 protein, and we will consider performing similar analysis on other NMD components in the future that may include UPF2 and SMG genes. In this study, different oncogenic mutations at the ATP-binding sites/substrate pocket of UPF1 and at its interface with UPF2 (CH-domain) were assessed using MD simulations, for their effects on the associated molecular properties and interactions, as well as on protein stability. Initial in silico stability screening of cancer associated mutations resulted in the identification of five promising and important mutations: K164R, R253W, T499M, E637, and E833K. We characterized these mutations for effects on stability as potential gain or loss-of-function attributes.

A first insight was obtained when comparing the structural stability of the wild-type and the mutant forms of the UPF1 protein in its apo state. The RMSD values for the apo wild-type, E637K, and E833K systems were higher compared to when complexed with AMPPNP, whereas the reverse was observed for T499M, K164R, and R253W mutant systems. In addition, residues in the CH-domain (between 116 and 295) of UPF1 that bind UPF2 were highly mobile (i.e., in RMSF) compared to any other domain of the UPF1 protein. Concerning the substrate pocket of the UPF1 protein, residues T499 and E833 have different RMSFs in UPF1 apo-form/AMPPNP bound and wild-type/mutant UPF1, whereas the mobility of the residue E637 remains the same (RMSF ~1 Å) in all these systems. Principal component analysis of the first eigenvector identified that the absence of UPF2 or AMPPNP (apo-state) drives the movement of the UPF1 CH-domain towards the helicase-domain. Moreover, in the UPF1 ATP-site mutants, motions of the CH-domain were observed for T499M and E833K complexed with AMPPNP compared to the apo-form, whereas mutant E637K showed the reverse behavior. Overall, the UPF1 CH-domain mutant systems obtained well defined clusters when complexed with UPF2 than in the apo-state. Such data also highlight the opportunity to take these dynamic conformational variations into consideration when developing drug screens that target various biochemical properties of the UPF1 protein.

Hydrogen bonding analysis provided additional insights into the likely mode of action of UPF1. This screen identified UPF1 substrate binding site mutant systems that have fewer H-bonds (~4) when in complex with AMPPNP, compared to the wild-type UPF1 (~8), whereas the CH-domain mutant systems (K164R/R253W) have increased UPF1-UPF2 interactions (~8–10/14–16 H-bonds) compared to the wild-type system (~5). In particular, mutation of residue at position 164 (K164R) in UPF1 enhanced its H-bond interactions with UPF2 that were absent in the wild-type system, and this interaction was enabled as a result of a conformational change of the UPF2 protein. The UPF1 residues S152, S203, S205, and Q230 formed H-bonds with UPF2 in all UPF1-UPF2 complexes, and the residue R703 formed H-bonds with AMPPNP in all UPF1-AMPPNP systems. As with RMSD values, insights into hydrogen bonding dynamics can be tested when developing new chemical biology tools/functional assays to investigate the functional dynamics of the UPF1 protein in the future such as hydrogen deuterium exchange mass spectrometry.

Finally, MDS data suggest a dominant impact of potential phosphorylation of the Serine residues in UPF1 at position 152, 203, and 205 that may modulate the interactions between UPF1 and UPF2. Furthermore, residues that formed interactions with AMPPNP in WT UPF1 system and at least one mutant system were: G495, T496, G497, K498, T499, K533, and G831. The mutated residues K637 and K833 formed H-bond interactions with AMPPNP, whereas the mutant M499 lacked such interactions with AMPPNP. The residue level contributions in binding energy (∆E) suggest that the UPF1 residues positioned at 637 and 833 contributed more binding energy to AMPPNP in their mutant forms (E637K and E833K) compared to their native state, while T499M had lower binding energy with AMPPNP compared to the wild-type. The MDS findings suggest that the side-chain of the T499M mutant prevents the tri-phosphate moiety of the AMPPNP molecule forming optimal interactions in the substrate binding site of UPF1, and this has the potential to affect the ATP-hydrolysis process of UPF1 or the NMD machinery.

## 4. Materials and Methods

### 4.1. System Setup

The crystal structures of UPF1 (PDB: 2WJY [10]) and UPF1-UPF2 (PDB: 2WJV [10]) were used to prepare the apo-form/-state of UPF1 and the complex, respectively. The structure of UPF1 bound to AMPPNP (ATP analogue, PDB: 2GJK [19]) lacks the CH-domain and therefore, the position of AMPPNP was used as a template to prepare AMPPNP bound to the ATP pocket of UPF1 with the CH-domain included (PDB: 2WJY [10]). Structures with unresolved residues as shown in Figure S1, were modeled using the “prepare protein” protocol implemented in the macromolecules module of BIOVIA Discovery Studio Client v18.1 program [Dassault Systemes, BIOVIA Corp., San Diego, CA, USA]. This protocol prepares the protein by inserting missing atoms in incomplete residues, models missing loop regions, deletes alternate conformations of residues (disorder), removes waters, standardizes atom names, and protonates titratable residues using the program pKa [37]. The protocol was applied with the CHARMM forcefield and to assign protonation states the following parameters were used: pH 7.4, protein dielectric constant 10, and ionic strength 0.145 mM. Protein structure preparation was followed by energy minimization using 1000 steps of the "smart minimizer" algorithm with a dielectric constant of 1.0 in BIOVIA Discovery Studio Client v18.1. The non-bonded list radius was set to 14 Å for electrostatic and van der Waals interactions, and the non-bond higher/lower cut-off distance was 12/10 Å, respectively. In addition, the RMS gradient was set to 0.1 in the minimization.

### 4.2. Residue Scanning/Site-Directed Mutagenesis

Mutant models were generated and predicted changes in stability were computed using the “residue scan” module from the Molecular Operating Environment (MOE; Chemical Computing Group Inc., Montreal, QC, Canada) program. For each studied structure of UPF1 (apo-form and with AMPPNP/UPF2), one residue was mutated at a time, and the resulting stability change (∆∆G) upon mutation was calculated. “Residue scan” in MOE was performed using LowModeMD ensemble, 10,000 search iterations, and the CHARMM27 forcefield. The stability of a mutant was computed as the relative binding free energy difference (∆∆G_bind_) between the mutant and wild-type (WT) UPF1 protein. In detail, ∆∆G is the relative thermostability of the mutation with respect to the wild-type UPF1 protein, calculated as the Boltzmann average of the relative stabilities of the ensemble. A negative ∆∆G points to a stabilizing mutation while a positive ∆∆G suggests a destabilizing mutation [38].

### 4.3. Molecular Dynamics Simulations

Molecular dynamics simulations were performed for the UPF1 protein in its wild-type forms (apo, UPF1-AMPPNP, and UPF1-UPF2), as well as for the mutant proteins with mutated residues K164R (located in the UPF1-UPF2 interface) and R253W (placed in the CH-helicase interface) in the CH-domain (apo-form and UPF1-UPF2 complex). In addition, MDS were also performed for the mutations in the ATP-binding site or the substrate pocket including T499M, E833K and E637K (apo-form and UPF1-AMPPNP complex), as these residues were speculated to modulate the ATP-binding or the hydrolysis process. The GROMACS 4.6.5 [39,40] program was used to perform MDS (for 13 model systems) by applying the Gromos96 43a1 forcefield [41]. For the AMPPNP molecule, the topologies were generated using the PRODRG server as it is compatible with the GROMACS program [42]. Moreover, Gaussian 09 (revision E.01) package [43] was used to optimize the geometries and to calculate the partial charges of AMPPNP. The charges were acquired by fitting the electrostatic potential to fixed charges using the “charges from electrostatic potentials” with a grid-based method (CHELPG). The obtained CHELPG charges for AMPPNP were suitable for MD simulations as they capture higher order effects arising from dipoles [44].

The prepared model systems/structures were solvated using a simple point charge (SPC) water molecules in a dodecahedron box (10 Å thick). Periodic boundary conditions were applied and the simulation systems were neutralized with the appropriate number of Na^+^ and Cl^-^ counter ions (corresponding to a physiological salt concentration of 150 mM; Figure S1C). To relax any steric clashes, the system was subjected to energy minimization through 50000 steps of the steepest descent algorithm. The particle mesh Ewald (PME) method [45] was used to treat long-range electrostatic interactions, and the LINCS algorithm [46] was used to constrain the bond lengths. The cutoff radii for van der Waals and Coulomb interactions were set to 10 Å. The system was subsequently equilibrated in an NPT (isobaric-isothermal) ensemble simulation for 1000 ps. Standard temperature (300 K) and pressure (1 bar) were maintained by applying a V-rescale thermostat [47] and a Parrinello–Rahman barostat [48], respectively. Finally, a production run was performed for 1000 ns using a leapfrog integrator [49]. The trajectories were saved every 10 ps and analyzed using GROMACS tools and VMD [50]. The cutoffs to define H-bonds were 3.5 Å for the donor-acceptor distance, and 160°–180° for the intermolecular angle between donor-H-acceptor atoms.

Binding energies (ΔG) were estimated by subjecting the MDS to molecular mechanics/Poisson–Boltzmann surface area (MM-PBSA) evaluations using the g_mmpbsa program [51]. Protein–protein or protein–ATP binding energies were calculated for snapshots sampled every 1 ns of the 1000 ns simulations for the UPF1-AMPPNP and UPF1-UPF2 complexes. Computing the binding energy using g_mmpbsa [51] excludes the entropy contribution. Furthermore, to study the global motion of wild-type and mutant UPF1 in apo-forms and UPF1-AMPPNP/UPF2 complexes, principal component analysis (PCA) was carried out. This method reduces the complexity of the data and extracts motions from the MD trajectory that are functionally significant [52]. The calculation of the eigenvectors and eigenvalues was carried out using the g_cover module of the GROMACS software utilities, by diagonalizing the covariance matrix, and the g_anaeig tool was used to analyze and plot the eigenvectors [53].

## 5. Conclusions

The cancer genomic sequencing data suggests that residues in the ATP-binding site and CH-domain (i.e., UPF2 interacting interface) of the UPF1 protein are frequently mutated, and we assessed the effects of those mutations on UPF1using in silico methodologies. The most promising key residues of UPF1 were identified by MDS and were in common (wild-type as well as mutant systems) in forming hydrogen bond interactions with UPF2 or AMPPNP as follows: S152, S203, S205, and Q230 or R703, respectively. Residues G495, T496, G497, K498, T499, K533, and G831 formed interactions with AMPPNP in wild-type and in at least one mutant system. Based on our findings, we suggest that phosphorylation of the Serine residues at position 152, 203, and 205 in UPF1 may modulate/control UPF1-UPF2 interactions/binding.

The CH-domain K164R and R253Wmutant systems present as well-defined conformational clusters when complexed with UPF2 compared to the apo-state, and have increased UPF1-UPF2 interactions compared to the wild-type system. Moreover, the E637K and E833K alleles in the UPF1 substrate binding site improved the affinity of AMPPNP for the UPF1 protein, whereas the T499M mutant hindered the tri-phosphate moiety of AMPPNP informing optimal interactions with UPF1.

Altogether, our analysis of the structural changes in different UPF1 systems suggests the flexible nature of the substrate binding region in UPF1 with a predictable impact by certain cancer-associated mutations. These in silico site-directed mutagenesis findings can be useful in understanding the molecular mechanisms of important NMD components (i.e., UPF1 and UPF2) as well as to guide future experimental studies that aim to develop chemical tools to impact on UPF1-dependent processes.

## Supplementary Materials

Supplementary materials can be found at https://www.mdpi.com/1422-0067/20/22/5644/s1.

## Abbreviations

NMD	nonsense-mediated mRNA decay
mRNAs	messenger RNAs
PTCs	premature termination codons
eRF1	eukaryotic release factor 1
UPF1	up frameshift 1
DHX34	DEAH box polypeptide 34
SMG1C	suppressor with morphological effect on genitalia 1 complex
EJC	exon junction complex
CCR4	carbon catabolite repressor protein 4
DCPC	decapping complex
PNRC2	pro-rich nuclear receptor co-activator 2
CH-domain	cysteine-histidine-rich domain
SF1	superfamily 1
nsSNPs	non-synonymous single nucleotide polymorphism
MDS	molecular dynamics simulations
MOE	Molecular Operating Environment
WT	wild-type
SPC	simple point charge
PME	particle mesh Ewald
MM-PBSA	molecular mechanics/Poisson-Boltzmann surface area
PCA	principal component analysis
P bodies	processing bodies
TCGA	the cancer genome atlas
RMSD	root mean square deviation
RMSF	root mean square fluctuation
Rg	radius of gyration
