# Supplementary material for: Insights into the Effects of Cancer Associated Mutations at the UPF2 and ATP-Binding Sites of NMD Master Regulator: UPF1
Source: Int J Mol Sci. 2019 Nov 11;20(22):5644. doi: 10.3390/ijms20225644 (PMC6888360; doi:10.3390/ijms20225644)
Supplement: Supplementary file 1 [file ijms-20-05644-s001.pdf]

Supporting Materials

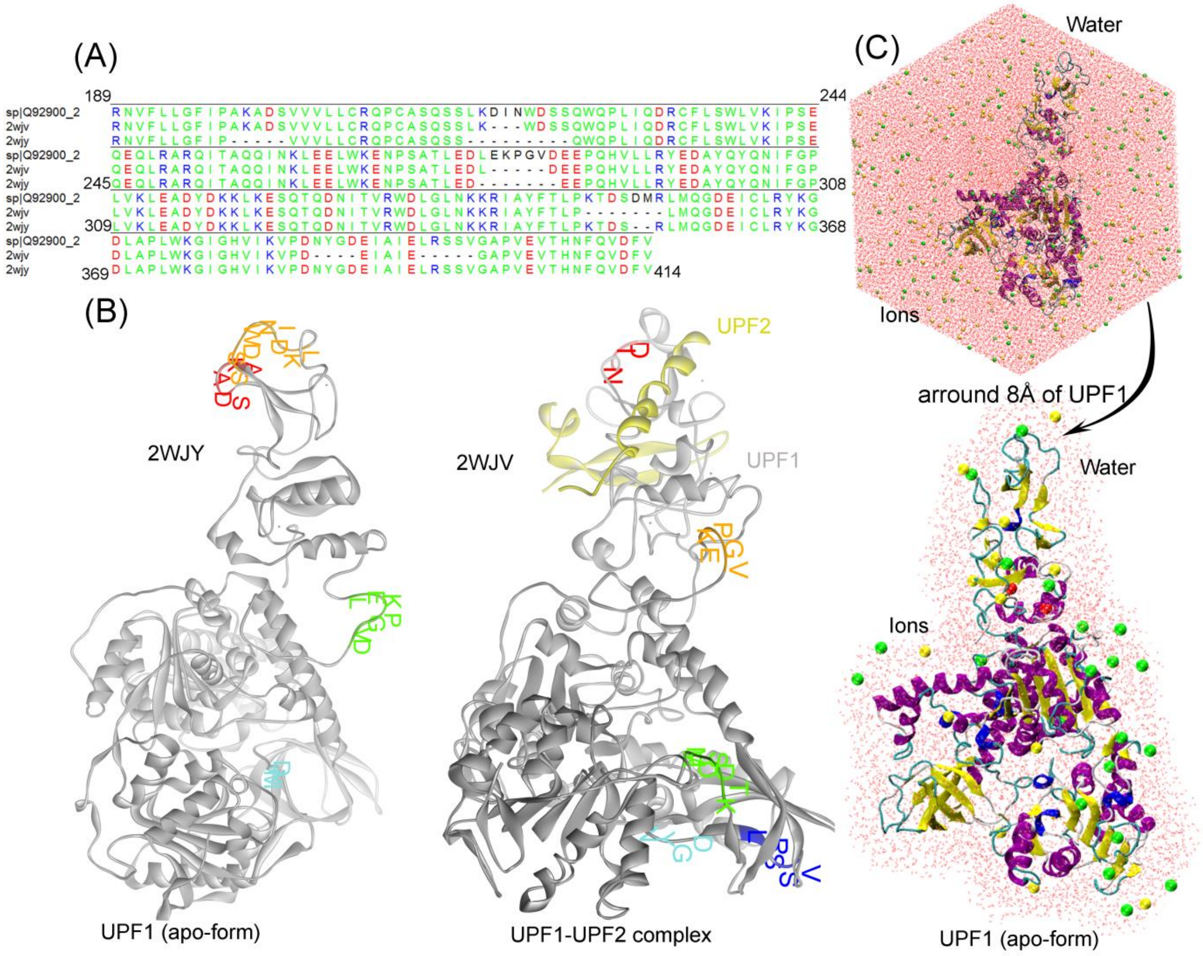

Figure S1. The UPF1 protein. **(A)** Structure-based sequence alignment of the UPF1 protein in the apo state (PDB: 2WJY) and in the UPF1-UPF2 complex (PDB: 2WJV). **(B)** Structures of the UPF1 protein with labelled missing residuesthat were inserted in the protein. **(C)** MDS system ofthe UPF1 apo-form; one cell in periodic boundary conditions (PBC) representing water and ions within 8 Å of the UPF1 protein.

Table S1. The UPF1 gene altered in different cancer types, data retrieved from the cBioPortal database (Cancer Discov.2012, 2, 401-404).

Cancer type	Frequency genes altered; cases (%)	Total cases
Breast cancer	89 (1.03%)	8653
Ovarian cancer	86 (6.38%)	1347
Non-small cell lung cancer	69 (0.88%)	7834
Endometrial cancer	59 (4.1%)	1440
Colorectal adenocarcinoma	48 (3.22%)	1489
Endometrial carcinoma	42 (7.17%)	586
Prostate cancer	40 (0.92%)	4354
Ovarian epithelial tumor	38 (6.5%)	585
Melanoma	38 (1.85%)	2058
Esophagogastric cancer	37 (1.59%)	2323
Prostate adenocarcinoma	26 (1.99%)	1304
Bladder cancer	26 (1.14%)	2282
Invasive breast cancer	23 (1.2%)	1918
Head and neck cancer	21 (1.39%)	1512
Esophagogastric adenocarcinoma	18 (3.04%)	592
Skin cancer, non-melanoma	17 (3.15%)	540

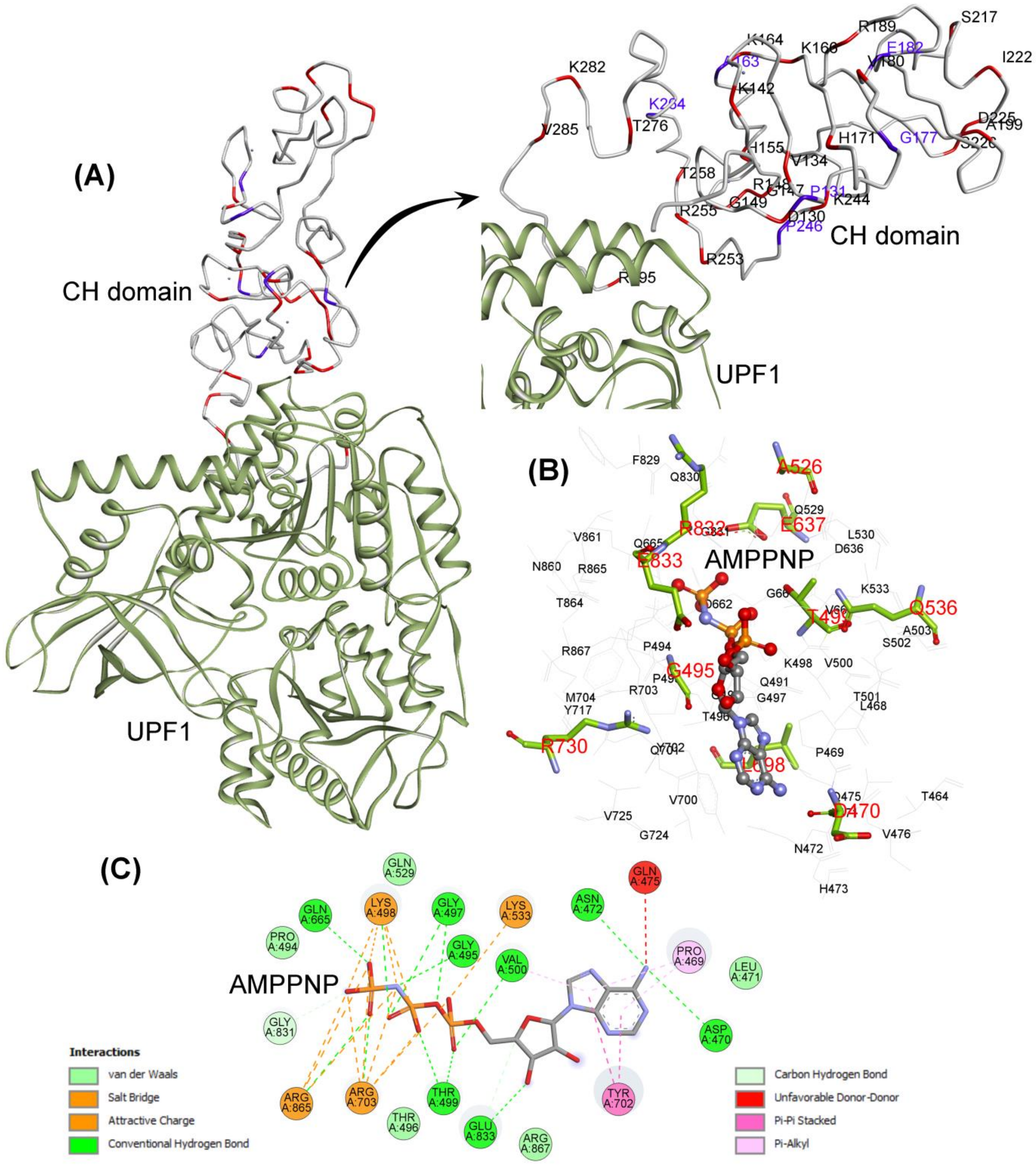

Figure S2. The UPF1 protein mutations analysis from the cBioPortal database (Cancer Discov.2012, 2, 401-404). **(A)** Residues from the CH-domain of the UPF1 protein that are mutated in different types of cancer (mutations with frequency ≥ 2 are in blue color). **(B)** UPF1 residues within 8 Å of AMPPNP (ATP binding site; PDB: 2GJK) are labelled, and residues found mutated in different cancer types are represented as stick (carbon atoms are colored in green) and labelled in red. (Color scheme: oxygen in red, carbon grey/green, hydrogen in silver, nitrogen in blue, and sulphur in orange). **(C)** 2D diagram representing different type of interactions between UPF1 and ATP analogue (AMPPNP).

Table S2. The intramolecular hydrogen bond interactions between CH-helicase domains of the UPF1 protein.Interactions with occupancy (Occup.) ≥20% are presented in this table.

**Apo**			**UPF1-UPF2 complex**			**UPF1-ATP complex**		
**CH**	**Helicase**	**Occup.**	**CH**	**Helicase**	**Occup.**	**CH**	**Helicase**	**Occup.**
Pro119	Thr429	52.59%	Leu252	Ala299	43.47%	Glu278	Tyr300	58.18%
Leu293	Tyr296	51.50%	Leu294	Arg687	39.18%	Gln256	Asn304	40.02%
Glu278	Gln303	46.51%	Gln256	Asp298	37.89%	Ala254	Asp298	39.42%
Asp279	Gln424	36.03%	Val292	Gly679	29.91%	Leu293	Gln682	38.92%
Arg253	Tyr300	27.74%	Arg253	Val437	29.61%	Leu252	Ala299	38.22%
Tyr125	Tyr300	27.64%	His291	Gly679	26.32%	Lys116	Thr429	24.55%
Arg253	Ala431	26.45%	Arg255	Asp298	20.94%	Leu293	Tyr296	24.25%
Arg253	Val437	24.75%	Glu278	Asn304	20.64%	Arg253	Ala431	20.86%
Glu278	Gln424	23.25%	Arg253	Tyr300	20.64%			

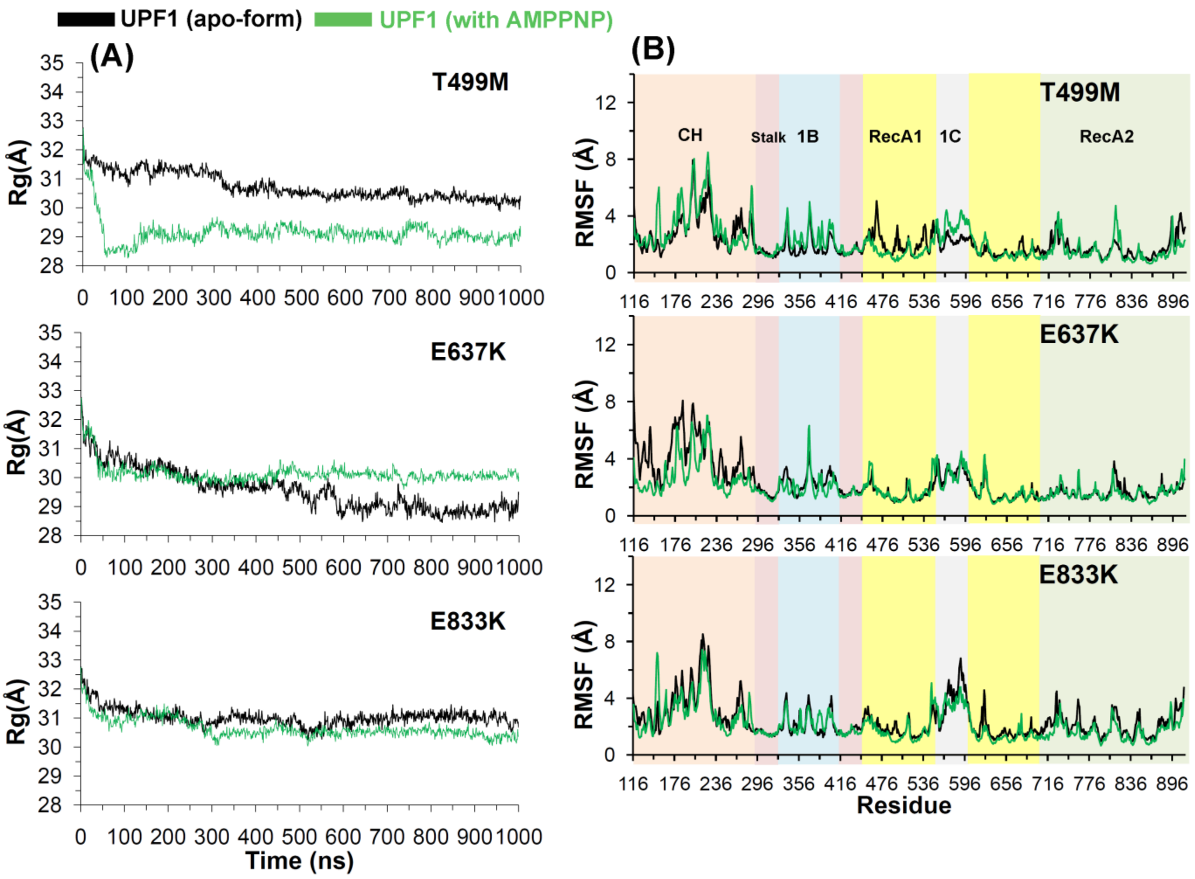

Figure S3. Structural analysis of the UPF1 ATP-site mutant systems: T499M, E637K, and E833K. **(A)** Radius of gyration profile (Rg), representing structural changes of UPF1 protein during the MD simulations. **(B)** RMSF analysis of the UPF1 protein showing fluctuations of residues in different simulated systems.

Table S3. Intermolecular H-bond interactions of the wild-type systems between UPF1-UPF2 or UPF1-AMPPNP, represented with the occupancy (Occup.) of a particular interaction over time frame (interactions ≥ 10 % occupancy are presented in this table).

**UPF1-UPF2**			**UPF1-AMPNP**	
**UPF1**	**UPF2**	**Occup.**	**UPF1**	**Occup.**
Val205	Val1172	61.71%	Thr499	74.75%
Ser203	Val1172	53.84%	Lys498	60.08%
Val205	Leu1174	51.15%	Gly495	58.98%
Ser152	Asp1110	44.37%	Arg703	27.05%
Asn190	Asn1124	33.10%	Thr496	17.86%
Ala201	His1198	27.42%	Lys533	17.07%
Gln260	Glu1109	23.13%	Gly497	15.67%
Gln230	Ala1195	21.64%	Arg730	12.97%
Gln234	Gln1193	20.04%	Gly831	12.77%
Ser152	Asp1110	19.64%		
Leu207	Leu1174	18.25%		
Glu248	Met1103	17.35%		
Glu248	Gly1104	16.35%		
Gln228	Asn1197	13.66%		
Ser240	Gln1193	11.27%		

Table S4. The intramolecular H-bond interactions between CH-helicase domains of the UPF1 protein from mutated systems: T499M, E637K, and E833K showing occupancy (Occup.) of particular interaction over time frame. Interaction ≥ 20 % occupancy are presented in this table.

**Apo-form**			**UPF1-ATP complex**		
**CH**	**Helicase**	**Occup.**	**CH**	**Helicase**	**Occup.**
**T499M**					
Gln256	Asp298	64.17%	Leu293	Tyr296	52.20%
Leu294	Arg687	44.21%	Glu278	Tyr300	50.00%
Leu252	Ala299	42.51%	Val292	Ala678	35.23%
Arg255	Asp298	42.32%	Tyr125	Tyr300	22.26%
Glu271	Tyr300	35.93%	Glu278	Gln303	21.06%
Leu293	Tyr296	29.14%	Arg253	Ala431	20.16%
Leu118	Thr429	27.45%			
Arg253	Ala431	24.75%			
Arg253	Tyr300	23.15%			
Glu248	Tyr442	21.56%			
Gln256	Glu297	21.16%			
**E637K**					
Gln256	Asp298	54.69%	Leu293	Tyr296	45.11%
Glu278	Gln303	47.01%	Arg253	Val437	24.45%
Glu281	Gln301	38.32%	Arg253	Tyr300	22.95%
Gln290	Lys677	28.14%	Asp279	Tyr300	22.65%
Val292	Gln682	28.04%	Gln256	Asp298	21.86%
His129	Tyr300	23.95%	Asp279	Gln303	20.86%
**E833K**					
Thr258	Glu297	63.67%	Tyr442	Leu252	47.70%
Leu293	Tyr296	57.88%	Gln256	Asp298	44.41%
Gln290	Asn304	52.30%	Leu293	Tyr296	39.12%
Thr151	Glu297	41.72%	Gln261	Asp298	34.13%
Asn304	Asp279	36.53%	Tyr316	Glu287	33.93%
Asn304	Pro283	34.13%	Gln682	Leu293	33.33%
Gln261	Asp298	32.34%	Lys674	Glu287	30.24%
Gln303	Asp279	31.84%	Leu277	Tyr300	23.45%
Arg255	Asp298	29.24%	Asp117	Asp433	22.65%
Tyr300	Glu278	27.84%	Tyr300	Glu278	17.86%

Table S5. The intermolecular H-bond interactions between UPF1-AMPPNP from mutated systems: T499M, E637K, and E833K with the occupancy (Occup.) of particular interaction over time frame (interaction ≥ 10 % occupancy are presented in this table).

UPF1-AMPPNP					
**T499M**		**E637K**		**E833K**	
**UPF1**	**Occup.**	**UPF1**	**Occup.**	**UPF1**	**Occup.**
Gly831	48.20%	Thr499	73.95%	Thr496	52.69%
Arg703	43.91%	Thr499	66.67%	Gly497	45.61%
Gln475	36.93%	Arg865	49.50%	Lys498	33.63%
Arg832	35.13%	Arg703	27.74%	Arg703	33.53%
Gln830	25.95%	Thr496	27.25%	Arg867	26.75%
Arg865	22.36%	Gly495	22.75%	Thr496	18.86%
Lys498	15.57%	Lys637	17.66%	Val500	12.97%
Gln529	13.77%	Gly497	16.37%	Lys833	12.38%
Asp470	10.88%	Lys533	10.88%	Gly495	12.18%

Table S6. The intramolecular H-bond interactions between the CH-helicase domain of UPF1 from mutant model systems: K164R and R253W, representing occupancy (Occup.) of particular interaction over time frame. Interactions ≥ 20 % occupancy are presented in this table.

**Apo-form**			**UPF1-UPF2 complex**		
**CH**	**Helicase**	**Occup.**	**CH**	**Helicase**	**Occup.**
**K164R**					
Gln261	Glu297	53.89%	Gln256	Asp298	63.61%
Leu293	Tyr296	49.00%	Gln261	Glu297	51.94%
Gln260	Glu297	44.41%	Thr258	Glu297	50.75%
Thr258	Glu297	42.12%	Gln261	Asp298	48.85%
Arg255	Asp298	41.12%	Leu293	Tyr296	48.65%
Arg253	Val437	40.42%	Leu280	Asn304	35.89%
Tyr300	Asp279	36.73%	Arg253	Ala431	35.19%
Asp130	Glu434	34.73%	Gln260	Glu297	31.61%
Ala259	Glu297	25.15%	Asp279	Tyr300	26.72%
Asn304	Asp279	23.35%	Val292	Gly679	26.22%
			Leu294	Arg687	25.92%
**R253W**					
Gln261	Asp298	49.05%	Gln260	Glu297	50.75%
Gln256	Asp298	45.46%	Gln261	Asp298	48.35%
Leu293	Tyr296	43.47%	Arg255	Glu434	44.27%
Gln290	Asn304	38.88%	Thr258	Asp298	43.67%
Leu252	Ala299	30.01%	Asn150	Glu297	38.58%
Glu278	Asn304	27.82%	Asp279	Tyr300	38.48%
Trp253	Tyr300	26.52%	Gln261	Gln301	20.84%
Thr258	Glu297	23.93%			
Gln261	Glu297	21.34%			
Glu278	Tyr300	21.14%			
Leu118	Asp433	21.14%			
Arg255	Asp298	20.14%			

Table S7. The intermolecular H-bond interactions between UPF1-UPF2 from mutated model systems: K164R and R253W, with their occupancy (Occup.) of particular interaction over time frame (interactions ≥ 10 % occupancy are presented).

K164R			R253W		
**UPF1**	**UPF2**	**Occup.**	**UPF1**	**UPF2**	**Occup.**
Ser152	Asp1110	56.33%	Thr151	Asp1110	65.20%
Val205	Val1172	52.74%	Ser152	Asp1110	55.63%
Val205	Leu1174	45.16%	Ser217	Arg1128	53.14%
Asn150	Met1103	36.89%	Val205	Val1172	52.94%
Val180	Lys1177	35.79%	Leu207	Leu1174	52.44%
Ser203	Val1172	35.29%	Ser203	Val1172	48.55%
Phe192	Asn1124	35.29%	Val205	Leu1174	48.26%
Val180	Thr1175	32.70%	Ser226	Arg1128	43.87%
Asn150	Cys1107	29.01%	Ser215	Arg1128	39.08%
Glu182	Lys1177	23.33%	Gln211	Asn1124	38.29%
Glu182	Gly1178	20.14%	Ser227	His1198	35.89%
Ser152	Glu1109	18.84%	Gln216	Arg1128	34.90%
Asn190	Asn1124	16.45%	Ser215	Gln1127	34.00%
Leu193	Asn1124	14.56%	Glu178	Gln1181	33.60%
Gln230	Leu1194	13.26%	Gln211	Gln1127	30.71%
Asn150	Glu1109	13.06%	Glu178	Asn1179	24.53%
Arg164	Gln1127	10.87%	Glu178	Lys1180	23.83%
Gln228	Ser1191	10.77%	Arg210	Gln1127	23.23%
			His448	Gly1104	23.03%
			Gln230	Ala1196	21.93%
			Lys172	Gln1181	20.34%
			Leu170	Gln1181	13.26%
			Glu178	Gln1182	11.57%
			Ser218	Gln1127	10.67%
			Ala259	Asp1110	10.67%

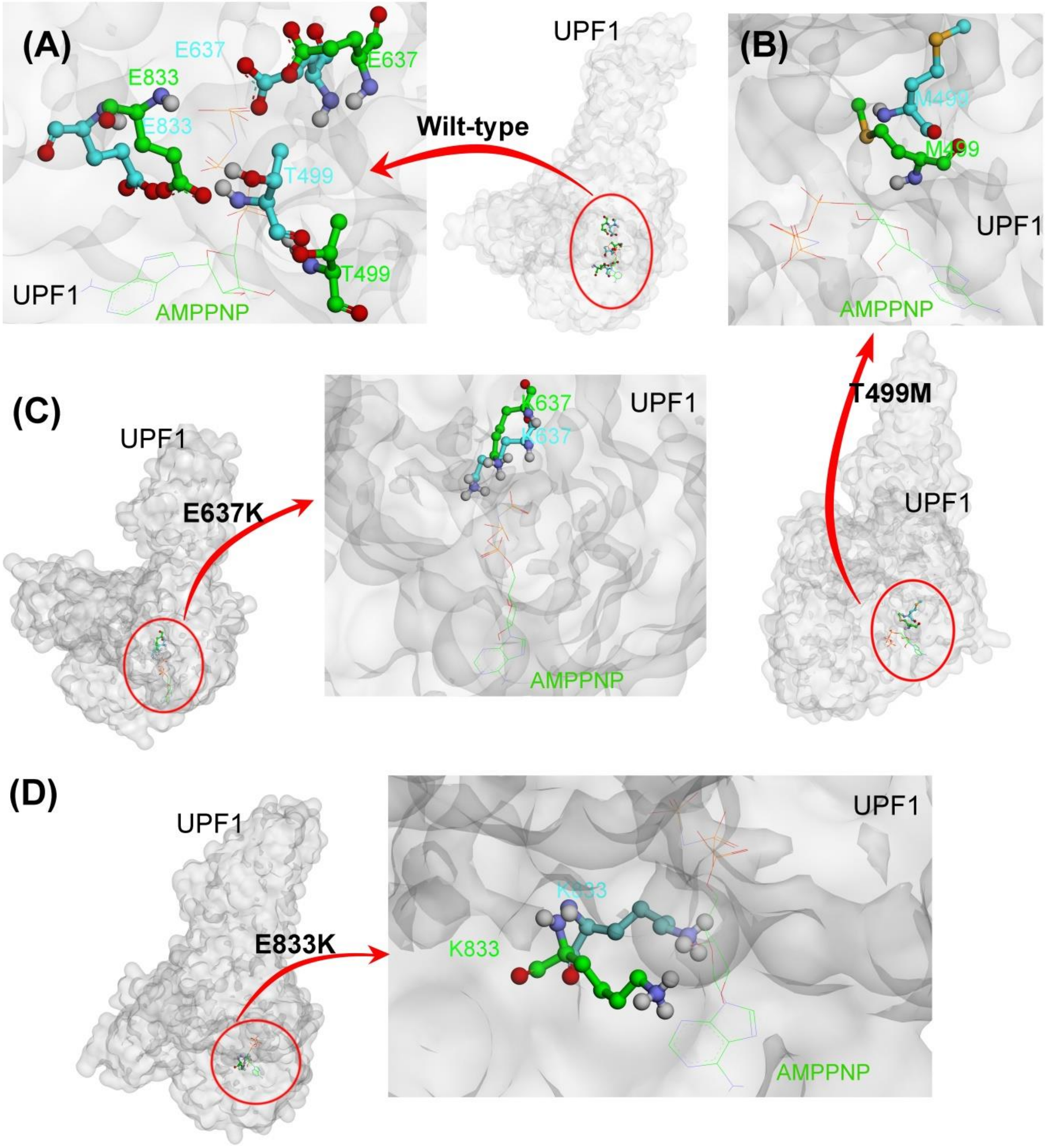

Figure S4. Binding mode of the AMPPNP molecule and the dynamics of residues in apo-form (blue color) and UPF1-AMPPNP complexes (green color) analysed from the trajectory of MD simulations for models:**(A)** wild-type, **(B)** T499M, **(C)** E637K, and **(D)** E833K. Color scheme: carbon in blue/green, oxygen in red, hydrogen in silver, nitrogen in blue, and sulphur in orange.

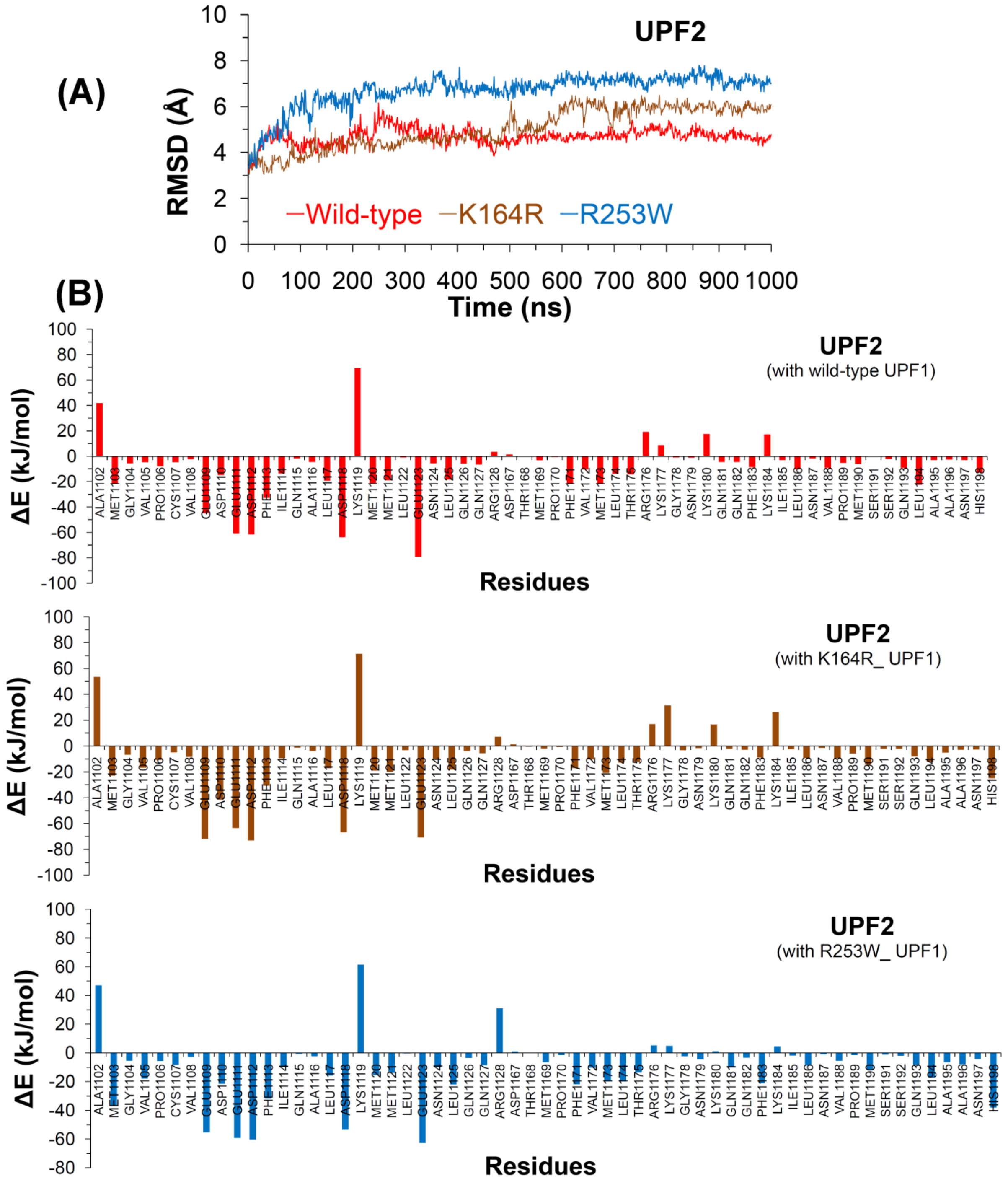

Figure S5. Structural analysis of the UPF2 protein from the wild-type/K164R/R253W UPF1-UPF2 complexes. **(A)** RMSDs representing structural changes of UPF2 protein. **(B)** Energy contribution of the UPF2 residues in binding with UPF1 computed using MM-PBSA.

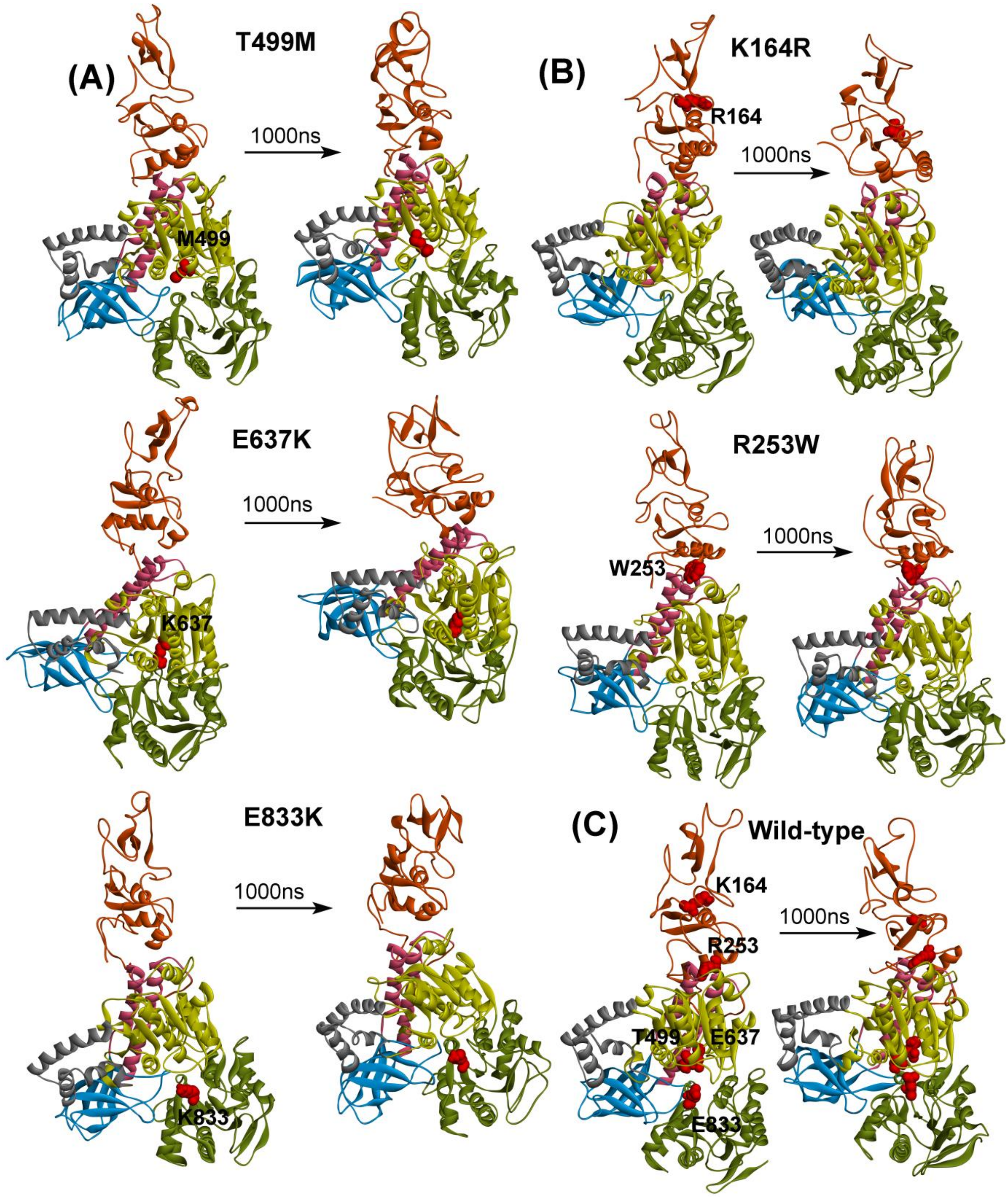

Figure S6. Structural changes of the UPF1 protein in apo-form obtained from the beginning and the end of MD simulations. **(A)** and (**B)** UPF1 ATP-site mutants T499M, E637K, and E833K; and UPF2-binding site mutants K164R and R253W. **(C)** Wild-type UPF1 protein conformation. Different domains of the UPF1 protein are colored as per Figure 1 in the main text.

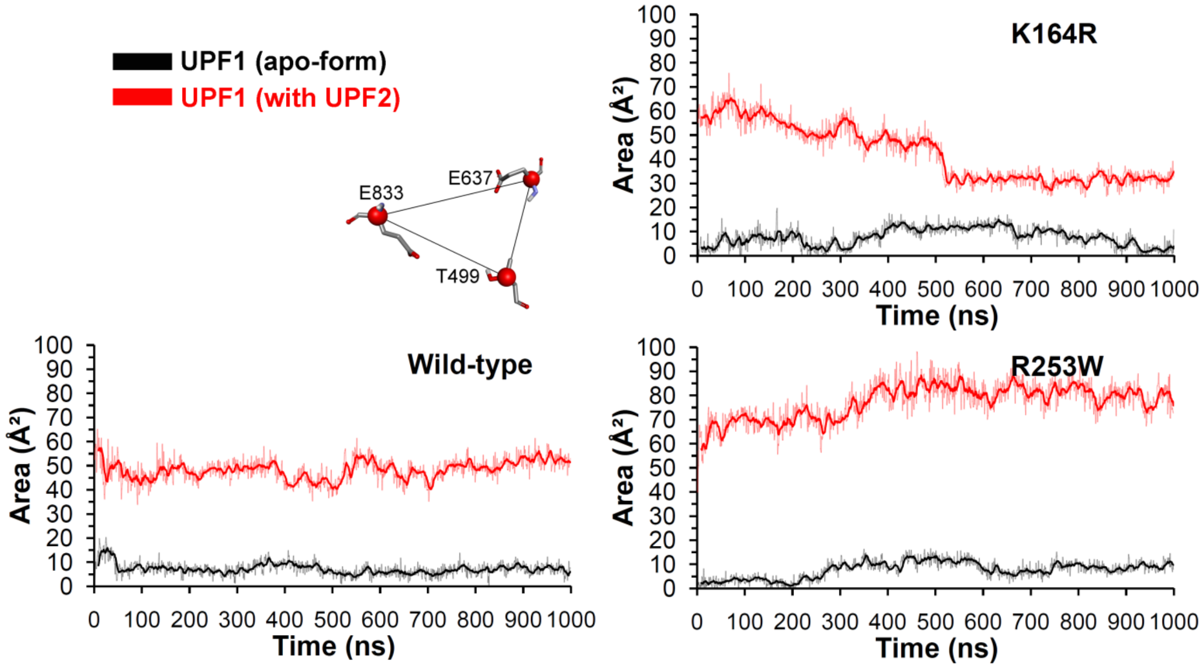

Figure S7. Area analysis of structural changes in the ATP-binding region of the UPF1 protein in wild-type, K164R, and R253W mutant model systems. The three residues (or triangle) selected for area calculation was based on Cα atoms of T499, E637, and E833 (black lines are for apo-form and red lines for UPF1-UPF2 complexes). The dark lines represents trend with a moving average of area with a period of 10 ns.

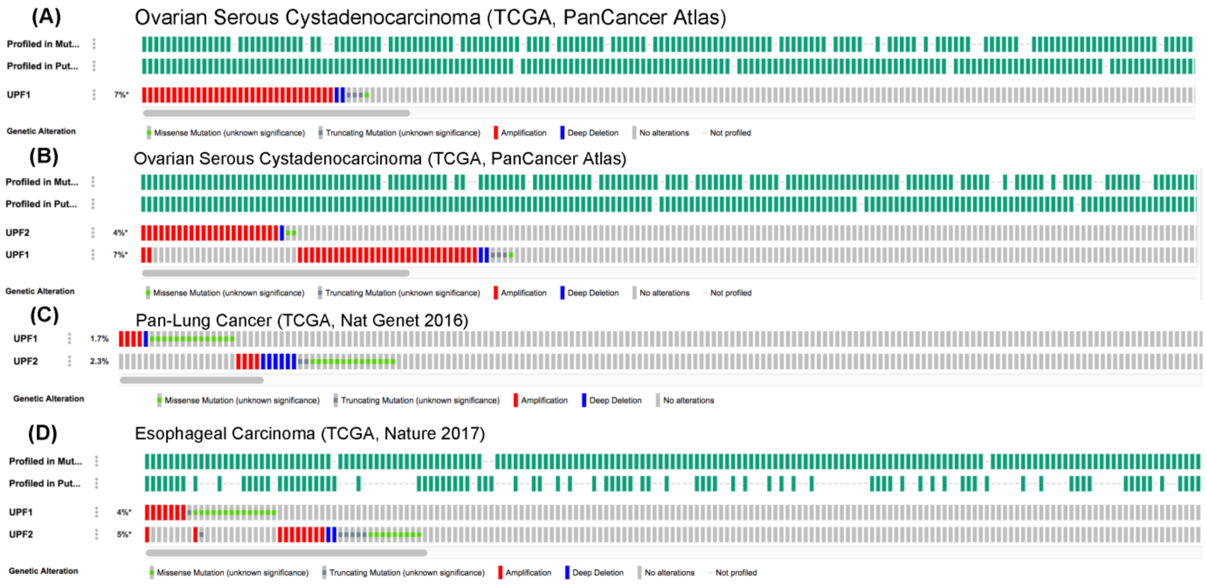

Figure S8. The UPF1 gene altered in ovarian cancer, lung cancer, and esophageal squamous cancer data retrieved from the cBioPortal database (Cancer Discov.2012, 2, 401-404). **(A)** UPF1 mutation frequency in ovarian serous cystadenocarcinoma (TCGA, PanCancer Atlas). **(B)** Amplification dominates for UPF1 or UPF2 in ovarian serous cystadenocarcinoma (TCGA, PanCancer Atlas). **(C)** Mutation dominates for UPF1 or UPF2 in pan-lung cancer (TCGA, Nat Genet 2016). **(D)** Mutation dominates for UPF1 or UPF2 in Esophageal Carcinoma (TCGA, Nature 2017).
